# Heme and iron toxicity in the aged spleen impairs T cell immunity through iron deprivation

**DOI:** 10.1038/s43587-025-00981-4

**Published:** 2025-10-17

**Authors:** David Ezuz, Heba Ombashe, Lana Watad, Akmaral Rakhymzhanova, Satyarth Pandey, Orna Atar, Esther G. Meyron-Holtz, Noga Ron-Harel

**Affiliations:** 1 Faculty of Biology, Technion- Israel Institute of Technology, Haifa, Israel; 2 Faculty of Biotechnology and Food Engineering, Technion- Israel Institute of Technology, Haifa, Israel

**Keywords:** Ageing, Metabolism, Lymphocytes, Cytokines

## Abstract

Mechanisms of T cell aging involve cell-intrinsic alterations and interactions with immune and stromal cells. Here we found that splenic T cells exhibit greater functional decline than lymph node T cells within the same aged mouse, prompting investigation into how the aged spleen contributes to T cell aging. Proteomic analysis revealed increased expression of heme detoxification in aged spleen-derived lymphocytes. Exposure to the heme- and iron-rich aged splenic microenvironment induced aging phenotypes in young T cells, including reduced proliferation and CD39 upregulation. T cells survived this hostile niche by maintaining a low labile iron pool, at least in part, via IRP2 downregulation to resist ferroptosis but failed to induce sufficient iron uptake for activation. Iron supplementation enhanced antigen-specific T cell responses in aged mice. This study identifies the aged spleen as a source of hemolytic signals that systemically impair T cell function, underscoring a trade-off between T cell survival and function and implicating iron metabolism in immune aging.

## Abstract (web summary)

In their study, Ezuz et al. show that the aged spleen contributes to T cell aging through hemolytic stress and an increasingly heme- and iron-rich microenvironment. T cells adapt by limiting their iron levels. This protective response impairs cell function but can be reversed in vivo by iron supplementation during activation.

## Main

T lymphocytes, the cellular arm of the adaptive immune response, protect the host against foreign pathogens and maintain tissue homeostasis. With aging, T cell functionality declines, leading to compromised immunity against infections, diminished response to vaccination and elevated susceptibility to autoinflammatory diseases and malignancies^1^. The mechanisms driving T cell dysfunction in aging involve universal hallmarks of cellular aging, including mitochondrial dysfunction, loss of proteostasis, genetic alterations and senescence^2^, together with T cell-specific hallmarks, including a reduction in the T cell repertoire and a phenotypic shift towards less naive and more differentiated cells in aged individuals^1^. Recent findings in mice show that the premature aging of the T cell compartment accelerates aging phenotypes in multiple organs^3,4^. Our study investigated this reciprocal interaction and identified the aged spleen as a source of hemolytic signals promoting T cell aging and dysfunction.

The spleen is organized in regions called the red pulp and white pulp, separated by an interface known as the marginal zone. The white pulp resembles the structure of a lymph node (LN), containing T cell and B cell zones, whereas the red pulp primarily functions to filter blood and recycle iron from senescent red blood cells (RBCs), a task predominantly carried out by red pulp macrophages^5^. Functional deterioration of red pulp macrophages with aging leads to accumulation of senescent RBCs, heme and iron deposits in the aged spleen^6^. Excess heme and iron could promote oxidative stress and lipid peroxidation^7^. We hypothesized that changes in the microenvironment of the aged spleen impact the T cell aging trajectory.

We show here that T cells extracted from the spleen displayed increased markers of aging and dysfunction, along with diminished viability and proliferative abilities relative to LN-derived T cells. Using comprehensive, whole-cell proteomics, we identified enhanced expression of proteins associated with stress and inflammation in naive T cells from aged versus young spleens, including enzymes involved in heme catabolism and iron storage. Subjecting young T cells to the aged spleen environment or to heme induced this unique protein profile and impeded their proliferation. To counter ferroptosis, a cell death pathway driven by iron and reactive oxygen species (ROS), T cells in the aged spleen adapted to oxidative conditions by limiting iron uptake and reducing labile iron pool. This adaptive response, however, compromised T cell proliferation, which could be restored through iron supplementation. Our findings reveal how adaptation of T cells to aging processes within their host tissue undermines their functionality.

## Results

### Exposure to the aged splenic microenvironment promotes T cell aging phenotypes

**Fig. 1:**
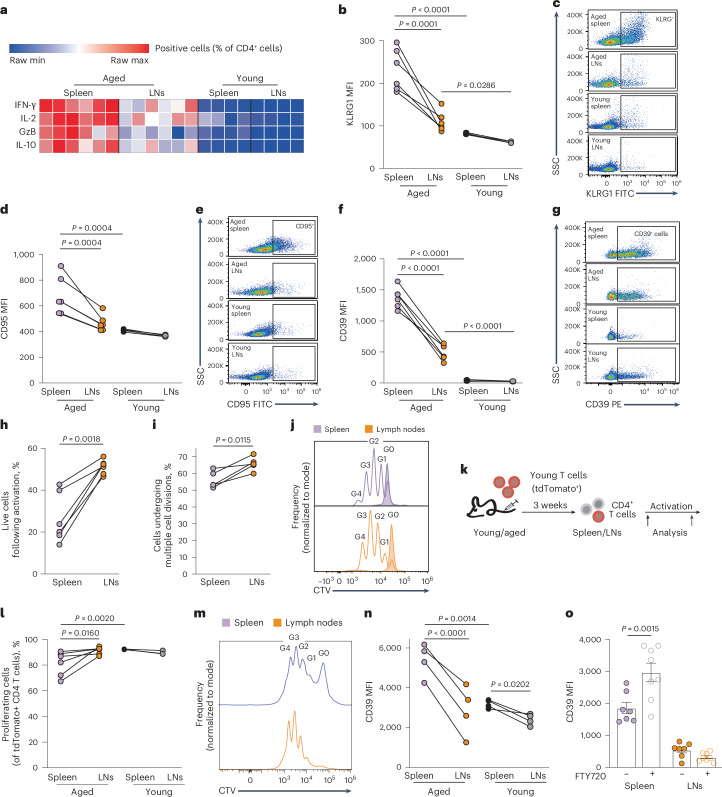
Exposure to the aged splenic microenvironment promotes T cell aging phenotypes. **a**–**g**, Bulk CD4^+^ T cells were purified from spleens and LNs of young (8–10 weeks old; *n* = 4) and aged (21–23 months old; *n* = 6) mice and analyzed by flow cytometry to quantify (**a**) cytokine production and expression of (**b,c**) KLRG1, (**d**,**e**) CD95 and (**f**,**g**) CD39. Naive CD4^+^ T cells (CD4^+^CD25^−^CD62L^+^CD44^lo^) were sorted from spleens and LNs of aged mice. **h**, Cell viability (*n* = 6) and (**i**,**j**) proliferation (*n* = 5) were quantified by flow cytometry after stimulation. **k**, Scheme showing experimental design. Young T cells derived from TdTomato^+^ transgenic mice were transfused into young or aged C57Bl/6 wild-type recipients. **l**–**n**, After 3 weeks, recipient mice were sacrificed, and CD4^+^ T cells from spleens and LNs were analyzed by flow cytometry, with and without activation, to quantify (**l**,**m**) proliferation (young: *n* = 3; aged: *n* = 6) and (**n**) CD39 expression (young: *n* = 4; aged: *n* = 4). **o**, Young TdTomato^+^ T cells were transfused into wild-type aged recipients, as in (**k**), followed by daily injections of FTY720 (*n* = 8) or vehicle (*n* = 7) for 2 weeks. CD39 expression was quantified on CD4^+^ T cells isolated from spleens and LNs. MFI, mean fluorescence intensity, calculated by geometric mean. *P* values calculated by two-way analysis of variance (ANOVA) with Sidak’s multiple comparisons test (**b**–**f**,**l**,**n**,**o**), or a two-tailed paired Student’s *t*-test (**h**,**i**). Each panel shows representative data from at least two independent experiments. Bar graphs represent mean ± standard error of the mean (s.e.m.). Data points represent single mice. Data points connected by a line signify samples collected from different lymphoid organs within the same mouse. Source data

**Extended Data Fig. 1:**
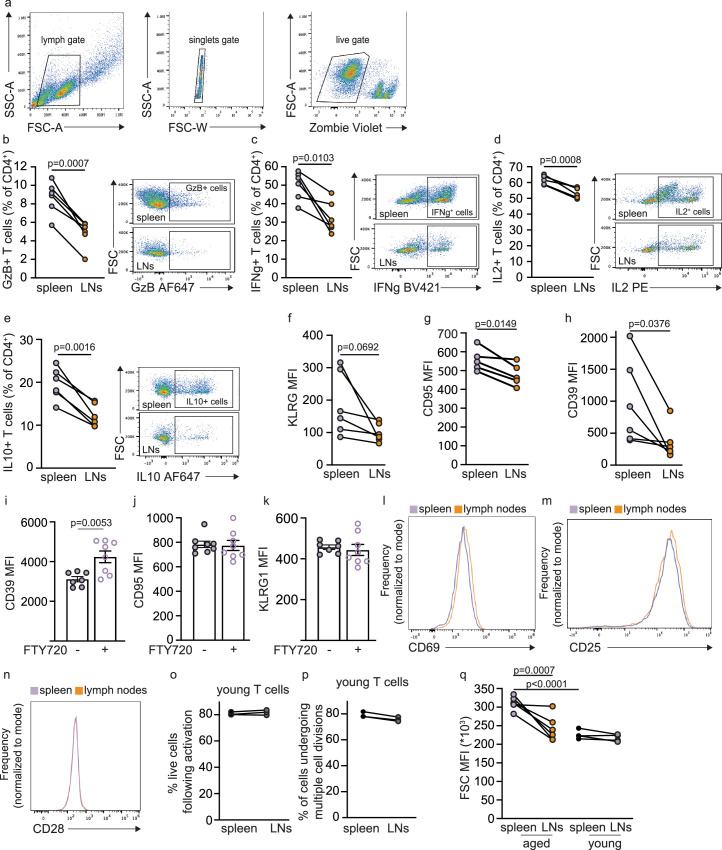
Exposure to the aged splenic microenvironment promotes T cell aging phenotypes. **a**) Plots demonstrating gating strategy for flow cytometry experiment. Bulk CD4^+^ T cells were purified from spleens and lymph nodes (LNs) of aged (21-23 month old; n = 6) mice and analyzed by flow cytometry to quantify production of (**b**) GzB, (**c**) IFNγ, (**d**) IL 2, and (**e**) IL-10. Expression of (**f**) KLRG1 (n = 6), (**g**) CD95 (n = 5), and (**h**) CD39 (n = 6) was analyzed gating on naïve CD4^+^ T cells (CD4^+^CD25^−^CD62L^+^CD44^l^°). (**i**-**k**) Aged mice were treated with FTY720 (daily injections, i.p.; n = 8) for 2 weeks. CD4^+^ T cells were analyzed for expression of (**i**) CD39, (**j**) CD95 and (**k**) KLRG1. Naïve CD4 + T cells were purified from spleens and LNs of aged mice, activated ex vivo and analyzed by flow cytometry to assess expression of the early activation markers (**l**) CD69 and (**m**) CD25, and (**n**) the co-stimulatory receptor CD28. Young CD4^+^ T cells (n = 3) collected from spleens and LNs were activated ex vivo. (**o**) Cell viability, and (**p**) proliferation were assessed. (**q**) Assessment of cell size in CD4^+^TdTomato^+^ T cells derived from the spleens and LNs of young (n = 4) and aged (n = 6) mice. Bar graphs show mean ± SEM. P values calculated by one-way ANOVA with Sidak’s multiple comparisons test (**q**), two-tailed paired (**b**-**h**,**o**,**p**), or two-tailed unpaired (**i**-**k**) Student’s t test. Each panel shows representative data from at least 2 independent experiments. Data points represent single mice. Data points connected by a line signify samples collected from different lymphoid organs within the same mouse. Source data

To determine the effects of aging on T cells in various immune organs, CD4^+^ T cells were isolated from the spleens and LNs of young (8–10 weeks old) and aged (21–23 months old) C57Bl/6 mice. The gating strategy used for all flow cytometry experiments is shown in Extended Data Fig. 1a. Compared to young T cells, aged T cells from both organs showed an elevation in production of cytokines, including granzyme B (GzB), interferon gamma (IFN-γ), interleukin-2 (IL-2) and IL-10, in agreement with previous studies^8^. However, cytokine production was significantly elevated in aged T cells collected from aged spleens compared to LNs from the same host (Fig. 1a and Extended Data Fig. 1b–e). Moreover, we detected elevation in multiple markers associated with T cell aging and senescence^8,9^, including KLRG1 (Fig. 1b,c), CD95 (Fig. 1d,e) and CD39 (Fig. 1f,g) on T cells collected from aged spleens. The composition of the T cell population changes with aging, with the accumulation of differentiated T cells and shrinkage of the naive T cell pool^1^. Nevertheless, expression of these markers was higher in T cells derived from aged spleens compared to those from LNs, even when gating specifically on naive T cells (CD4^+^CD25^−^CD62L^+^CD44^lo^; Extended Data Fig. 1f–h). Notably, CD39 expression on aged T cells increased even further when their ability to exit the spleen was blocked using FTY720, an inhibitor of sphingosine-1 phosphate^10^ (Extended Data Fig. 1i). Expression of KLRG1 and CD95 remained constant (Extended Data Fig. 1j,k). It is possible that CD95 and KLRG1, which mark terminally differentiated and exhausted T cells, could take longer to further increase, whereas CD39 expression reflects a dynamic response to the microenvironment.

To investigate how conditions in the aged spleen impact T cell functions, naive CD4^+^ T cells (CD4^+^CD25^−^CD62L^+^CD44^lo^) were purified from the spleens and LNs of aged mice and activated ex vivo using plate-bound anti-CD3 and anti-CD28. Significant functional differences were identified, as aged T cells collected from spleens showed lower viability (Fig. 1h) and reduced proliferative capacity (Fig. 1i,j), compared to aged T cells isolated from LNs. Expression levels of early activation markers (CD69, CD25) and the central co-stimulatory molecule (CD28) were not substantially different (Extended Data Fig. 1l–n). Importantly, a similar analysis comparing T cells from spleen and LNs of young mice demonstrated no differences in functionality (Extended Data Fig. 1o,p). These results raised the question of whether exposure to the aged splenic milieu was sufficient to induce these aging phenotypes. To address this question, young T cells (tdTomato^+^) were transfused intravenously into young or aged recipients, collected and analyzed after 3 weeks (Fig. 1k). Like aged T cells, young tdTomato^+^CD4^+^ T cells collected from aged spleens proliferated less than T cells purified from the LNs of the same hosts or from a young host upon activation (Fig. 1l, m). Furthermore, young Td-tomato^+^ CD4^+^ T cells collected from aged spleens showed a marked increase in cell size (Extended Data Fig. 1q) and elevated CD39 levels (Fig. 1n). Treatment with FTY720 further enhanced CD39 expression on young Td-Tomato^+^ T cells residing in aged spleen (Fig. 1o). Together, these results show that exposure to the aged splenic microenvironment exacerbated multiple phenotypes associated with T cell aging.

### T cells in the aged spleen express high levels of proteins closely associated with stress and inflammation

**Fig. 2:**
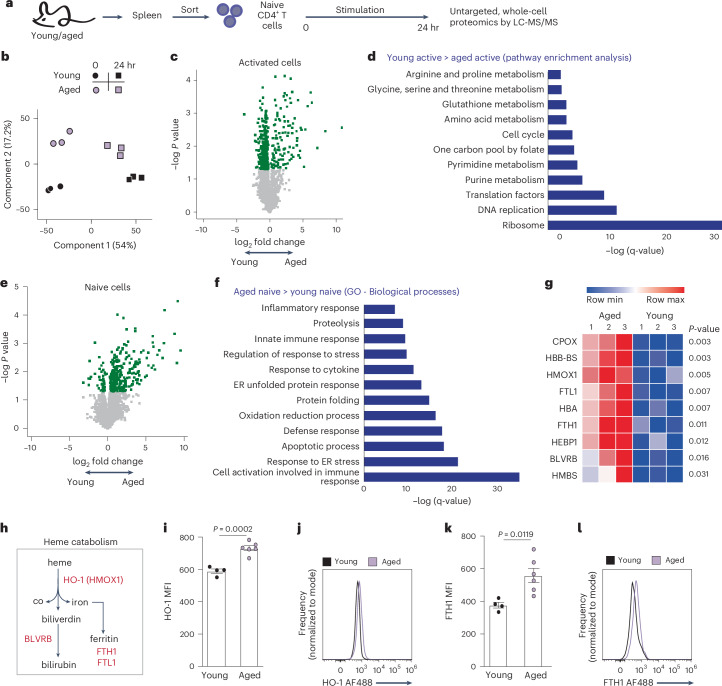
T cells in the aged spleen express high levels of proteins closely associated with stress and inflammation. **a**, Experimental scheme. Naive CD4^+^ T cells were sorted from the spleen of young (*n* = 3) and aged mice (*n* = 3 pools of five mice) and were either immediately frozen or stimulated ex vivo before protein extraction and digestion. The peptide pool from each sample was analyzed by LC-MS/MS. **b**, PCA. **c**, Volcano plot showing differences in protein levels between activated young and aged T cells. Green dots signify statistically significant results (two-tailed Student’s *t*-test *P* < 0.05). **d**, Pathways enriched among proteins significantly overrepresented in young versus aged T cells following activation. **e**, Volcano plot showing differences in protein levels between naive young and aged T cells. Green signifies statistically significant results (two-tailed Student’s *t*-test *P* < 0.05). **f**, Pathways enriched among proteins significantly overrepresented in aged versus young naive T cells. ER, endoplasmic reticulum; GO, Gene Ontology. **g**, Heatmap summarizing proteins associated with heme metabolism and detoxification, elevated in aged naive T cells compared to young cells. **h**, Key proteins involved in heme catabolism. **i**–**l**, Expression of HO-1 (**i**,**j**) and FTH1 (**k**,**l**) in naive CD4^+^ T cells collected from spleens of young (*n* = 4) and aged mice (*n* = 6), analyzed by flow cytometry. MFI was calculated by geometric mean. Bar graphs represent mean ± s.e.m. Data points represent single mice. *P* values were calculated by two-tailed unpaired Student’s *t*-test. Pathway enrichment analysis was performed using GSEA software. Pathways with a false discovery rate q value < 0.05 were considered significantly enriched. Source data

**Extended Data Fig. 2:**
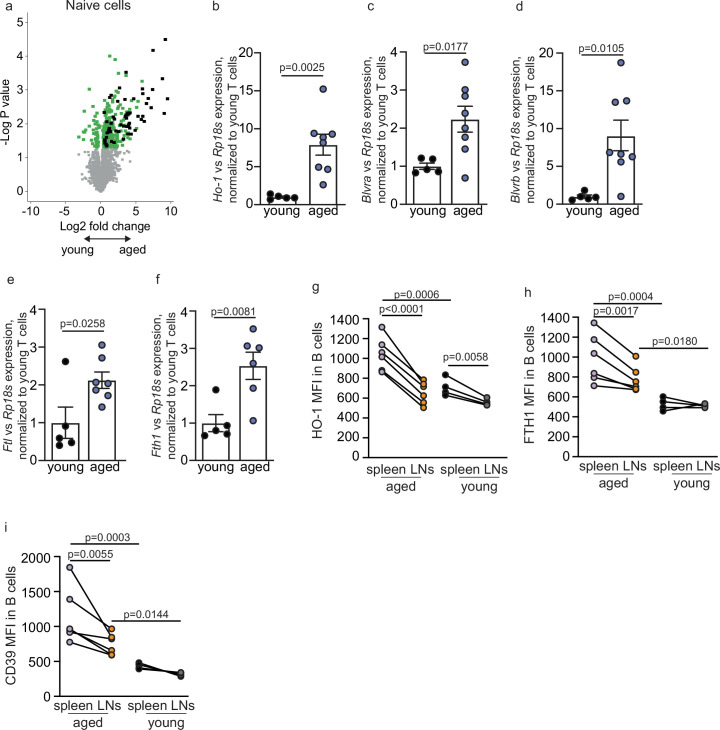
Lymphocytes in aged spleens upregulate expression of proteins associated with heme and iron detoxificaiton. (**a**) Volcano plot showing differences in protein levels between naive young and aged T cells. Green dots indicate statistical significance (Two-tailed Student’s t-test p value < 0.05). Black dots indicate proteins that remained significantly higher in activated aged compared to young T cells. (b-f) CD3^+^ T cells were isolated from the spleens of young and aged mice and analyzed by real-time qPCR to measure expression of genes encoding proteins involved in heme detoxification: (**b**) *Ho-1* (young: n = 5; aged: n = 8), (**c**) *Blvra* (young: n = 5; aged: n = 8), (**d**) *Blvrb* (young: n = 5; aged: n = 8), (**e**) *Ftl* (young: n = 5; aged n = 7) and (**f**) *Fth1*(young: n = 5; aged: n = 6). (**g**-**i**) B cells from spleens and LNs of young (n = 4) and aged (n = 6) mice were analyzed by flow cytometry to quantify expression of (**g**) HO-1, (h) FTH1, and (**i**) CD39. Bar graphs show mean ± SEM. P values calculated by Two-way ANOVA with Sidak’s multiple comparisons test (**g**-**h**), or Two-tailed unpaired Student’s t test (**b-f**). Each panel shows representative data of at least 2 independent experiments. Data points represent single mice. Data points connected by a line signify samples collected from different lymphoid organs within the same mouse. Source data

To identify the cellular response of T cells to the microenvironment in aged spleens, we performed whole-cell, label-free proteomic analysis of pure naive CD4^+^ T cells collected from the spleens of young and aged mice. Cells were immediately processed or stimulated ex vivo for 24 h before protein extraction, peptide degradation and analysis by liquid chromatography tandem mass spectrometry (LC-MS/MS)(Fig. 2a). The dynamic changes of over 3,800 proteins were determined (Supplementary Data File 1). Principal-component analysis (PCA) revealed that activation accounted for 54% of the variance (PCA1), whereas age accounted for 17.2% of the variance (PCA2; Fig. 2b). Accordingly, 692 proteins were differentially expressed between young and aged T cells following activation. Among these, 84% were higher in young T cells (Fig. 2c) and enriched for enzymes involved in multiple metabolic pathways, consistent with previous reports showing metabolic defects in aged T cells^11,12^ (Fig. 2d). We further identified enrichment for proteins involved in DNA replication and protein translation (Fig. 2d), consistent with the deficient growth and proliferation of aged T cells (Fig. 1). In the naive subpopulation, 330 proteins were differentially expressed between young and aged cells (Fig. 2e). Of these, 87% were overrepresented in aged T cells and enriched with proteins closely associated with activation, inflammation and stress (Fig. 2f). Most proteins overexpressed in resting aged T cells compared to their young counterparts remained elevated in aged T cells even after activation (Extended Data Fig. 2a), suggesting that aged T cells undergo enduring changes. Pathway enrichment analysis of the top 100 differentially expressed proteins, identified proteins associated with heme metabolism and degradation (Fig. 2g). Heme is catabolized by heme oxygenase 1 (HMOX1 or HO-1) to generate carbon monoxide (CO), biliverdin and labile iron. Excess iron is stored in cells within the cavity of ferritin, a globular hollow protein composed of 24 subunits of two types: ferritin H (FTH1) and ferritin L (FTL). Biliverdin is further reduced to bilirubin by biliverdin reductase (BLVRB) (Fig. 2h). These proteins were significantly elevated in aged naive CD4^+^ T cells compared to young cells (Fig. 2g). Overexpression of HO-1 (Fig. 2i,j) and ferritin (Fig. 2k,l) was further verified by flow cytometry.

We postulated that all T cells residing in the aged spleen were similarly exposed to these age-related signals. Indeed, qPCR analysis comparing bulk CD3^+^ T cells from the spleens of young and aged mice was performed, indicating increased expression of *Ho-1*, the two genes encoding for biliverdin reductase isozymes, *Blvra* and *Blvrb*, and the two ferritin subunits, *Ftl* and *Fth1* in aged T cells (Extended Data Fig. 2b–f). Thus, T cells within the aged spleen population expressed high levels of proteins involved with heme detoxification. A similar expression pattern seen in B cells (Extended Data Fig. 2g–i) further supports the notion of age-related changes in the splenic microenvironment affecting its lymphocytes.

### Hemolytic signals originating in aged spleens expose T cells to toxic heme and iron deposits

**Fig. 3:**
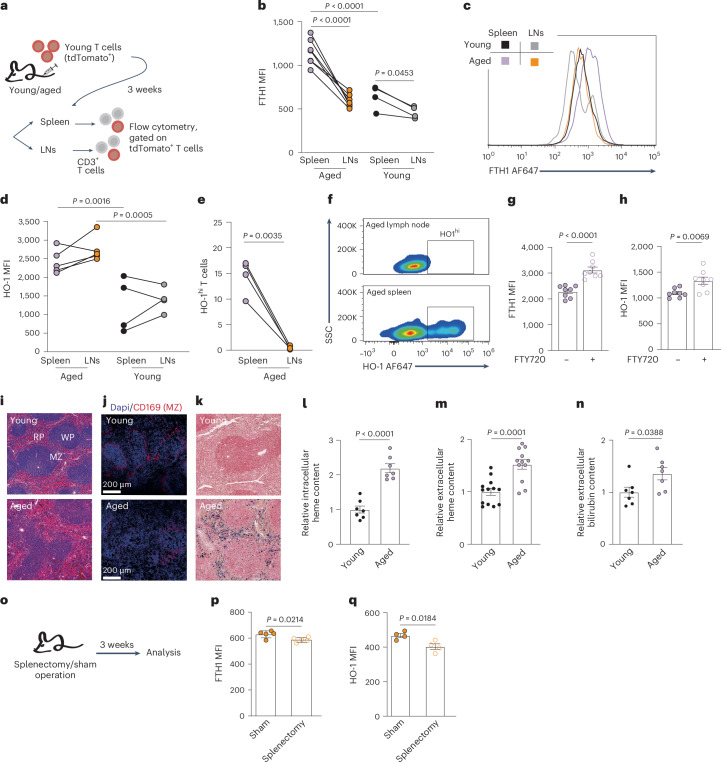
Hemolytic conditions in aged spleens expose T cells to toxic heme and iron deposits. **a**, Experimental scheme. Young T cells derived from TdTomato^+^ transgenic mice were transferred into young or aged C57Bl/6 wild-type recipients. After 3 weeks, CD3^+^ T cells were purified from the spleen and LNs and analyzed by flow cytometry to quantify (**b**,**c**) FTH1 expression (young: *n* = 4; aged: *n* = 7) and (**d**–**f**) HO-1 expression (young: *n* = 4; aged: *n* = 5 for panel **d** and *n* = 4 for panel **e**). **g**,**h**, In a similar adoptive transfer setup followed by a 2-week administration of FTY720, expression of (**g**) FTH1 and (**h**) HO-1 was quantified in spleen-derived T cells (*n* = 8). **i**, H&E processed paraffin sections of spleens derived from aged and young mice (RP, red pulp; WP, white pulp; MZ, marginal zone). **j**, Representative images of frozen spleen sections stained with anti-CD169 to mark marginal zone macrophages. **k**, Spleen paraffin sections from aged and young mice, stained with Prussian Blue to detect ferric iron deposits. **l**–**n**, Quantification of heme and bilirubin levels in young and aged spleens: (**l**) intracellular heme content in CD3^+^ T cells (*n* = 7), (**m**) heme levels in interstitial-fluid-enriched fraction (SE; young: *n* = 13; aged: *n* = 12) and (**n**) SE bilirubin levels (n = 7). (**o**), Experimental design. Aged mice underwent splenectomy or sham operation. After 3 weeks, CD3^+^ T cells were isolated from LNs. **p**–**q**, FTH1 (*n* = 5) and HO-1 (*n* = 4) levels were quantified by flow cytometry. MFI was calculated by geometric mean. Bar graphs represent mean ± s.e.m. *P* values were calculated by two-way ANOVA with Sidak’s multiple comparisons test (**b**,**d**), or two-tailed Student’s *t-*test (**e**,**g**,**h**,**l**–**n**,**p**,**q**). Each panel shows representative data of at least two independent experiments. Panels in **l**–**n** show pooled data from three independent experiments. Data points represent single mice. Data points connected by a line signify samples collected from different lymphoid organs within the same mouse. Source data

**Extended Data Fig. 3:**
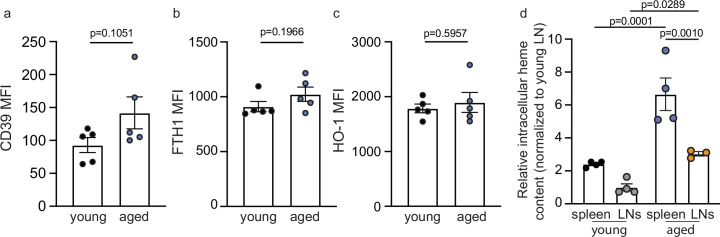
Hemolytic signals originating in aged spleens expose T cells to toxic heme and iron deposits. Young T cells derived from TdTomato^+^ transgenic mice were transfused into young (n = 5) or aged (n = 5) C57Bl/6 wild-type recipients. After 2 days, recipient mice were sacrificed, and CD3^+^ T cells from spleens and LNs were analyzed by flow cytometry to quantify expression levels of: (**a**) CD39, (**b**) FTH1, and (**c**) HO-1. (**d**) Spleens and LNs were excised from young (n = 4) and aged (spleen: n = 4; LNs: n = 3) mice. Intracellular heme content in purified CD3^+^ T cells was quantified. MFI: Mean Fluorescence Intensity, calculated by geometric mean. Bar graphs show mean ± SEM. Data points represent single mice. P values calculated by Two-tailed unpaired Student’s t-test (**a**-**c**) or Two-way ANOVA with Sidak’s multiple comparisons test. Source data

To directly test whether exposure to an aged splenic microenvironment in vivo was sufficient to induce the protein signature of aged T cells (Fig. 2), we repeated the adoptive transfer experiment of young tdTomato^+^ T cells into young or aged recipients (Fig. 3a). Strikingly, young TdTomato^+^ T cells in the aged spleen but not LNs significantly upregulated the FTH1 subunit of ferritin (Fig. 3b,c). HO-1 fluorescence intensity was elevated in T cells collected from aged compared to young hosts (Fig. 3d), and the percentage of T cells expressing high levels of HO-1 was higher among those collected from spleens compared to LNs in the same host (Fig. 3e,f). Notably, administration of FTY720 for 3 weeks significantly increased FTH1 and HO-1 expression in young TdTomato^+^ T cells retained in aged spleens (Fig. 3g,h), underscoring the spleen’s contribution to the observed phenotype. Nevertheless, brief 48 h exposure to an aged host environment was not sufficient to induce CD39, FTH1 or HO-1 expression in young TdTomato^+^ T cells (Extended Data Fig. 3a–c). With aging, the spleen’s architecture deteriorates, leading to a loss of clear boundaries between the red and white pulps^13^. This phenotype was confirmed with H&E staining and immunohistochemical analysis of CD169, a marker for marginal zone macrophages (Fig. 3i,j). Additionally, Prussian blue staining revealed elevated tissue iron deposits (Fig. 3k), consistent with previous studies^6^.

To investigate whether aged T cells are indeed exposed to elevated heme concentrations in vivo, we next quantified heme levels in T cells and within the splenic microenvironment. CD3⁺ T cells isolated from aged spleens exhibited significantly higher intracellular heme levels compared to those from young spleens (Fig. 3l), as well as compared to T cells from aged LNs (Extended Data Fig. 3d). Complementary analyses revealed increased levels of extracellular heme (Fig. 3m) and unconjugated bilirubin, a byproduct of heme degradation (Fig. 3n), in the interstitial fluid-enriched fraction from aged spleens relative to young controls.

Some phenotypes induced by the aged spleen microenvironment, including elevated expression of CD39 and HO-1, were also observed in T cells from aged LNs, though less prominently than in spleens (Figs. 1b,f and 3d). Similarly, intracellular heme levels in T cells from aged LNs were elevated relative to their young counterparts (Extended Data Fig. 3d). These findings suggest that although the spleen serves as the primary source of hemolysis-related signals, T cells in LNs are exposed to these signals as well, through systemic connection between the two lymphoid organs. To test this hypothesis, aged mice underwent either splenectomy or sham operations, with LNs collected 3 weeks after surgery for T cell purification and flow cytometric analysis (Fig. 3o). Splenectomy led to a mild but significant reduction in FTH1 and HO-1 (Fig. 3p,q) expression. In sum, heme and iron accumulation in aged spleens impacts T cell phenotype and function both locally and systemically, though less markedly in distal lymphoid organs.

### Heme induces cell death and promotes multiple aging-associated phenotypes in young T cells

**Extended Data Fig. 4:**
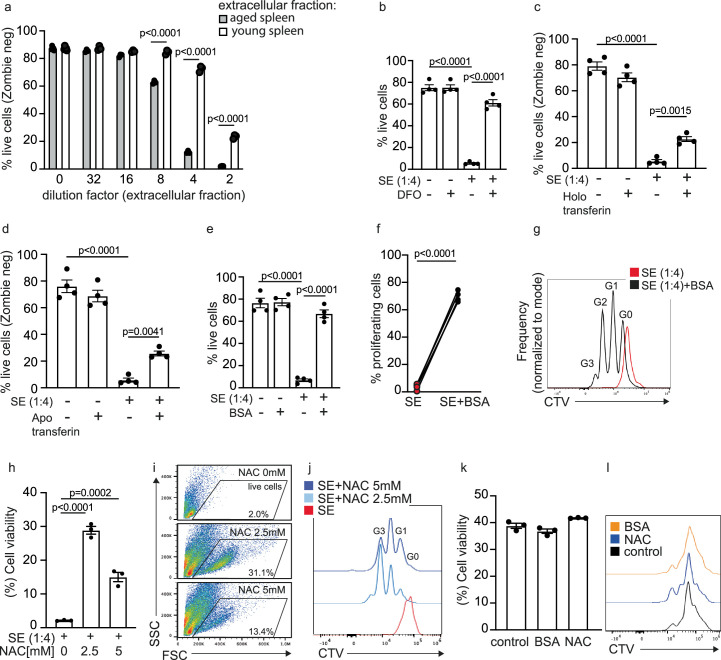
Interstitial fluids from aged spleens induce cell death and arrest proliferation in young T cells. (**a**) Young T cells (derived from a pool of 3 mice) were cultured ex vivo in media supplemented with different doses of interstitial-fluid-enriched fraction (SE) from young or aged spleens. Cell survival was quantified by flow cytometry using the Zombie reagent. (**b**-**e**) T cells derived from young mice (n = 4) were cultured in media supplemented with SE from aged spleens (1:4 dilution) with and without (**b**) DFO, (**c**) iron-saturated transferrin (holo-transferrin), (**d**) iron-deficient transferrin (apo-transferrin) or (**e**) BSA. Cell viability was quantified using the Zombie reagent. (**f**,**g**) Young T cells (n = 4) were stimulated in media supplemented with aged SE (1:4 dilution) ± BSA. Proliferation was assessed by flow cytometry. (**h**-**j**) Young T cells (n = 3) were treated with SE (1:4 dilution) in the presence of N-acetyl cysteine (NAC). (**h**,**i**) Cell viability and (**j**) proliferation were assessed by flow cytometry. (**k**,**l**) T cells from aged mice (n = 3) were stimulated in the presence of BSA or NAC to assess (**k**) cell viability and (**l**) proliferation. Each data point represents data collected from a single mouse, except for (**a**). Bar graphs show mean ± SEM. P values calculated by Two-way (**a**) or one-way ANOVA (**b**-**e**, **h**,**k**) with Sidak’s multiple comparisons test, or two-tailed, unpaired Student’s t test (**f**). Source data

To examine how T cells respond to the aged spleen milieu, CD3^+^ T cells purified from young mouse spleens were cultured ex vivo. Addition of interstitial fluid enriched fraction (SE) from aged spleens induced dose-dependent cell death. Notably, the cells survived significantly better in young SE (Extended Data Fig. 4a). Cell viability was rescued by deferoxamine (DFO), an iron chelator also known by its ability to bind iron aggregates and heme^14^ (Extended Data Fig. 4b). Partial rescue of cell viability with iron-saturated transferrin (Extended Data Fig. 4c) and unbound transferrin (Extended Data Fig. 4d), both with similar heme-binding capacity^15^, supported the contribution of heme and iron to T cell death under these conditions. Bovine serum albumin (BSA), a carrier of many circulating molecules including heme and its byproducts^16^, rescued cell death, to an extent comparable to DFO (Extended Data Fig. 4e). In addition to its effect on cell viability, SE completely abrogated T cell proliferation, and was neutralized by addition of BSA (Extended Data Fig. 4f,g). Together these findings underscore heme and iron as potential mediators of T cell dysfunction under hemolytic conditions.

**Fig. 4:**
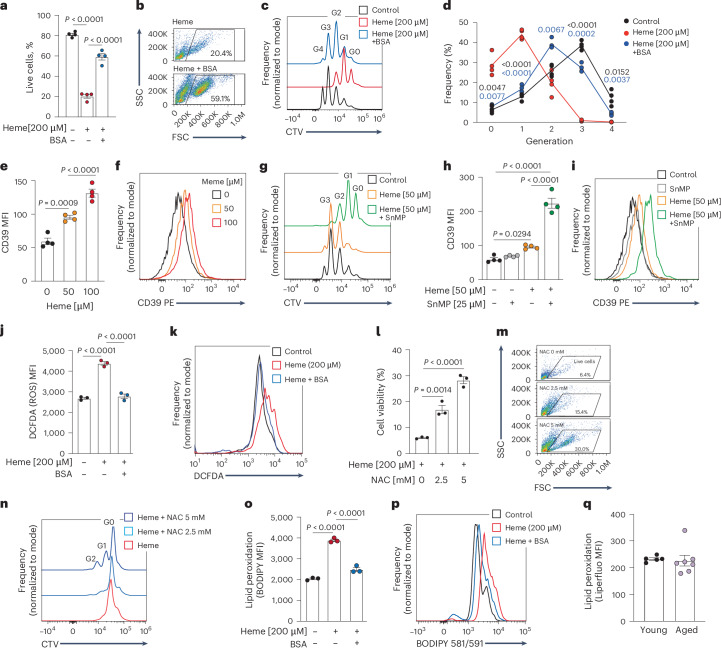
Heme induces cell death and promotes aging-like phenotypes in young T cells. **a**–**f**, T cells from young mice (*n* = 4) were activated in the presence of heme ± BSA and analyzed by flow cytometry to assess (**a**,**b**) cell viability, (**c**,**d**) proliferation (Gn indicates number of cell divisions) and (**e**,**f**) CD39 expression. **g**–**i**, Analysis of proliferation (**g**) and CD39 expression (**h**,**i**) in T cells from young mice (*n* = 4) activated in the presence of heme ± tin protoporphyrin IX (SnMP), an HO-1 inhibitor. **j**,**k**, Dichlorodihydrofluorescein diacetate (DCFDA) was used to quantify ROS in young T cells (*n* = 3) exposed to heme ± BSA for 48 h. Young T cells (*n* = 3) were activated in the presence of heme ± NAC for 48 h and analyzed by flow cytometry to assess (**l**,**m**) viability and (**n**) proliferation. **o**,**p**, Quantitation of BODIPY fluorescence intensity, an indicator of lipid peroxidation, in young T cells (*n* = 3) treated with heme ± BSA. **q**, Lipid peroxidation assessed using the Liperfluo reagent in young (*n* = 5) and aged (*n* = 7) T cells. MFI was calculated by geometric mean. Bar graphs represent mean ± s.e.m. *P* values were calculated by one-way ANOVA with Sidak’s multiple comparisons test (**a**,**e**,**h**,**j**,**l**,**o**), two-way repeated measure ANOVA with Tukey’s multiple comparison’s test (**d**) or two-tailed unpaired Student’s *t-*test (**q**). Each panel shows representative data of at least two independent experiments. Data points represent single mice. Source data

To directly test the impact of heme exposure on T cells, young CD3^+^ T cells were stimulated ex vivo, in the presence of heme, with or without BSA. Cell viability and proliferation were reduced by heme, and partially rescued by BSA (Fig. 4a–d). Moreover, exposure to heme was sufficient to induce CD39 in young T cells (Fig. 4e,f). To directly attribute these phenotypes to heme, and not to the products of its degradation (bilirubin, CO)^17,18^, T cells were treated with heme in combination with tin-mesoporphyrin IX (SnMP), an HO-1 inhibitor. SnMP caused proliferation arrest even under a lower dose of heme (50 μM; Fig. 4g) and led to an even greater increase in CD39 levels (Fig. 4h,i). These findings highlight heme’s contribution to multiple phenotypes acquired by T cells exposed to the aged spleen milieu. Heme induces ROS^19^. Accordingly, young T cells exposed to heme showed an increase in cellular ROS, which was completely abolished by BSA (Fig. 4j,k). Addition of the antioxidant *N*-acetyl cysteine (NAC) improved cell viability and proliferation of T cells treated with heme (Fig. 4l–n) or with SE (Extended Data Fig. 4h–j), suggesting that heme-induced aging phenotypes in young T cells were partly mediated by ROS. ROS promotes lipid peroxidation and cell death by ferroptosis^7^. Accordingly, lipid peroxidation was elevated in T cells exposed to heme and rescued by BSA (Fig. 4o,p). Taken together, these results suggested that heme accumulation in the aged spleen microenvironment induces oxidative stress in T cells and could lead to cell death by ferroptosis. Yet, to our surprise, neither BSA nor NAC improved survival or proliferation in aged T cells (Extended Data Fig. 4k,l). Moreover, quantification of lipid peroxidation in aged T cells compared to their young counterparts showed no apparent difference (Fig. 4q). Thus, heme exposure induces ROS production, lipid peroxidation, cell death, and proliferation arrest in young T cells. Notably, some of these phenotypes can also be triggered by iron, which is also present in the splenic environment. Yet, aged T cells seem to be resistant to lipid peroxidation and are able to survive in vivo. These results suggest that aged T cells develop mechanisms to tolerate the hostile microenvironment of the aged spleen.

### T cells residing in aged spleens develop resistance to ferroptosis

**Fig. 5:**
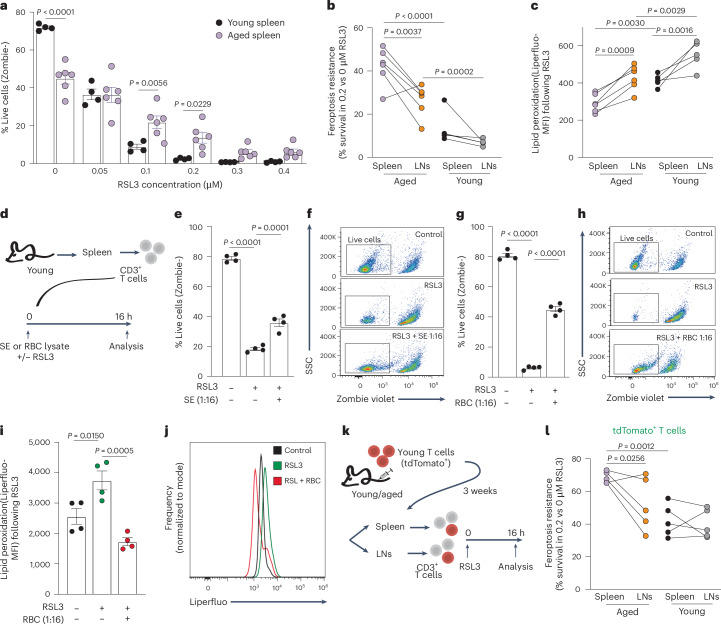
T cells residing in aged spleens develop resistance to ferroptosis. **a**, T cells purified from spleens of young (*n* = 4) or aged (*n* = 6) mice were treated with increasing concentrations of RSL3. Cell survival was quantified using the Zombie reagent, by flow cytometry. **b**,**c**, T cells collected from spleens and LNs of young (*n* = 5) and aged (*n* = 6) mice were treated with RSL3 to assess (**b**) ferroptosis resistance (calculated as the relative viability of T cells treated with 0.2 μM vs 0μM RSL3) and (**c**) lipid peroxidation (using Liperfluo dye). **d**, Experimental scheme: young CD3^+^ T cells (*n* = 4) were treated with RSL3 ± interstitial-fluid-enriched fraction (SE) from aged spleens or RBC lysate, for 16 h. **e**–**h**, Analysis of cell viability. **i**,**j**, Quantitation of lipid peroxidation. **k**, Schematic of experimental design. Adoptive cell transfer was performed as described in Fig. 1k. Purified T cells from young (*n* = 5) and aged (*n* = 5) aged recipients were cultured for 16 h with RSL3 before analysis by flow cytometry. **l**, Ferroptosis resistance of transferred TdTomato^+^ T cells, analyzed as the relative viability in 0.2 μM versus 0 μM RSL3. MFI was calculated by geometric mean. Bar graphs represent mean ± s.e.m. *P* values calculated by Two-way ANOVA (**a**–**c**,l) or one-way ANOVA (**e**,**g**,**i**) with Sidak’s multiple comparisons test). Each panel shows representative data of at least two independent experiments. Data points represent single mice. Data points connected by a line signify samples collected from different lymphoid organs within the same mouse. Source data

**Extended Data Fig. 5:**
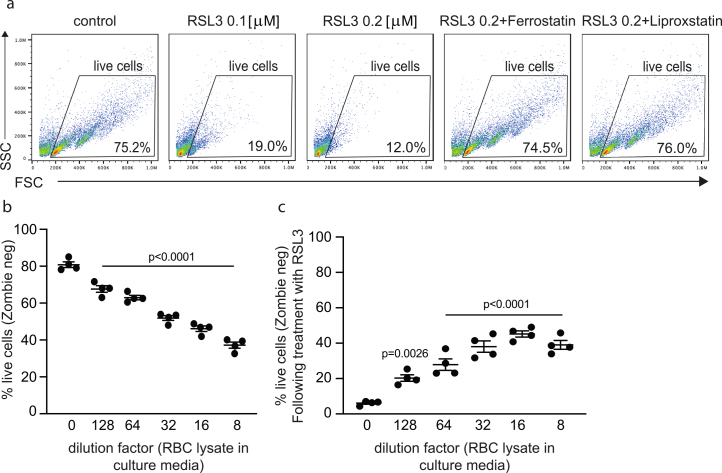
RBC lysate promotes ferroptosis resistance in young T cells. (**a**) Young T cells were cultured in media containing the indicated concentrations of RSL3 ± Ferrostatin or Liproxstatin, commercially available inhibitors of ferroptosis. Representative FACS plots gated for “live cells” are presented. (**b**) Young T cells (n = 4) were cultured in media containing red blood cell (RBC) lysate at the indicated concentrations. Cell viability was determined by flow cytometry using the Zombie reagent. (**c**) An analogous experiment as in (**b**), with the addition of RSL3 to culture media. Each data point represents data collected from a single mouse. Error bars show mean ± SEM. P values calculated by One-way ANOVA with Sidak’s multiple comparisons test, comparing all treatments to control cells. Source data

To survive in an aged spleen, T cells would need to develop resistance to ferroptosis. To demonstrate this effect, CD3^+^ T cells were isolated from spleens of young and aged mice and treated with RSL3 ((1S,3 R)-RSL3), which induces ferroptosis by inhibition of glutathione peroxidase 4 (ref. ^20^). As expected, exposure to RSL3 promoted cell death in young T cells and was rescued by ferrostatin-1 or liproxstatin-1, two commercially available inhibitors of ferroptosis (Extended Data Fig. 5a). Strikingly, aged T cells (from spleen or LNs) survived RSL3 treatment better than T cells from young mice, even at high RSL3 concentrations (Fig. 5a,b). Notably, T cells derived from aged spleens showed significantly better survival following RSL3 treatment compared to those from the LNs of the same donors (Fig. 5b). In both age groups, RSL3-induced lipid peroxidation was lower in T cells from spleens compared to those from LNs. In line with their superior ferroptosis resistance, T cells from aged spleens showed the lowest levels of lipid peroxidation (Fig. 5c). These findings were recapitulated ex vivo, as young T cells exposed to aged SE (1:16 in culture media, a concentration that did not reduce viability (Extended Data Fig. 4a)) resisted RSL3-induced ferroptosis better than cells treated with RSL3 alone (Fig. 5d–f). We postulated that many compounds in SE originate from RBC hemolysis in aged spleens. T cells exposed to RBC lysate in culture, showed a dose-dependent reduction in viability (Extended Data Fig. 5b). However, cells that survived this treatment were resistant to RSL3-induced ferroptosis (Fig. 5g,h and Extended Data Fig. 5c) and lipid peroxidation (Fig. 5i,j). These data support our hypothesis that RBC are the source of the signals promoting ferroptosis resistance.

To test whether exposure to the spleen microenvironment in vivo induced ferroptosis resistance in T cells, we performed adoptive cell transfer of TdTomato^+^ T cells from young donors into young or aged recipients. The response of TdTomato^+^ T cells to RSL3 was analyzed after 3 weeks (Fig. 5k). Strikingly, the T cells most resistant to ferroptosis were those derived from aged spleens (Fig. 5l). Together, these results show that T cells exposed to the milieu of the aged spleen adapt to their microenvironment by acquiring resistance to ferroptosis.

### Aged T cells resist ferroptosis by limiting labile iron pool via downregulation of IRP2

**Fig. 6:**
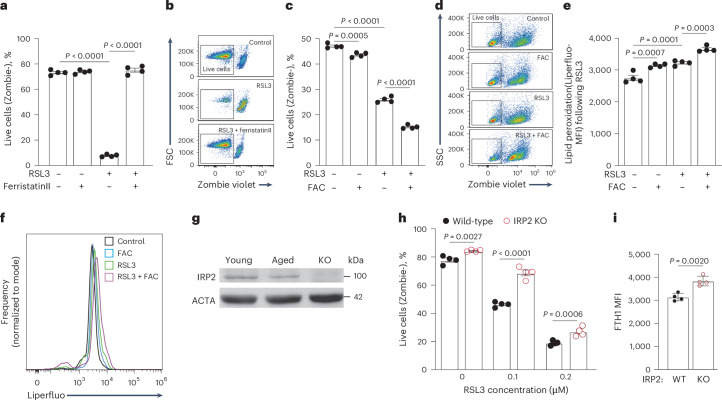
Aged T cells resist ferroptosis by limiting labile iron pools via downregulation of IRP2. **a**,**b**, Young T cells (*n* = 4) were treated with RSL3 ± Ferristatin II. Cell viability was assessed by Zombie staining. Aged T cells (*n* = 4) were treated with RSL3 ± FAC. **c**–**f**, Cell viability (**c**,**d**) and lipid peroxidation (**e**,**f**) were analyzed. **g**, IRP2 protein levels were quantified by immunoblot in splenic T cells isolated from young (*n*=pool of 4 mice) and aged (*n*=pool of 6 mice) mice. T cells from IRP2 KO mice (*n* = 4) were used as control. **h**,**i**, Resistance to RSL3-induced ferroptosis (**h**) and FTH1 MFI (**i**) were evaluated in T cells from IRP2 wild-type (n = 4) and KO (*n* = 4) mice. MFI: Mean Fluorescence Intensity, calculated by geometric mean. Bar graphs represent mean ± s.e.m. *P* values were calculated by two-tailed Student’s *t*-test (**i**), one-way ANOVA (**a**,**c**,**e**) or two-way ANOVA (h) with Sidak’s multiple comparisons test. Each panel shows representative data of at least two independent experiments. Data points represent single mice. Source data

One mechanism by which cells resist ferroptosis is by restriction of labile iron pool^21^. Accordingly, young T cells treated with ferristatin II, an iron uptake inhibitor, became resistant to RSL3-induced ferroptosis (Fig. 6a,b). To determine if aged T cells use the same mechanism for ferroptosis resistance, they were exposed to RSL3, and supplemented with ferric ammonium citrate (FAC). FAC eliminated ferroptosis resistance to RSL3 (Fig. 6c,d) and induced lipid peroxidation (Fig. 6e,f). Cellular iron levels are regulated by iron regulatory proteins 1 and 2 (IRP1 and IRP2). When iron levels are high, IRPs detach from iron regulatory elements (IREs) on mRNAs, enabling the translation of ferritin for iron storage, and the degradation of mRNAs encoding iron transporters including transferrin receptor 1 (TFR1, also known as CD71) to limit iron uptake^22^. Under such conditions, IRP2 undergoes FBXL5-mediated degradation^23^. We hypothesized that exposure to hemolytic conditions in aged spleens results in IRP2 downregulation in T cells. Indeed, IRP2 protein levels were reduced by twofold in aged compared to young splenic T cells (Fig. 6g), in agreement with elevated ferritin levels measured in these cells (Fig. 3). Consistent with these findings, young IRP2-deficient T cells were significantly more resistant to ferroptosis than control T cells (Fig. 6h) and contained more ferritin (Fig. 6i).

### Exposure to hemolytic conditions suppresses CD71 and iron uptake during T cell activation to avoid ferroptosis

**Fig. 7:**
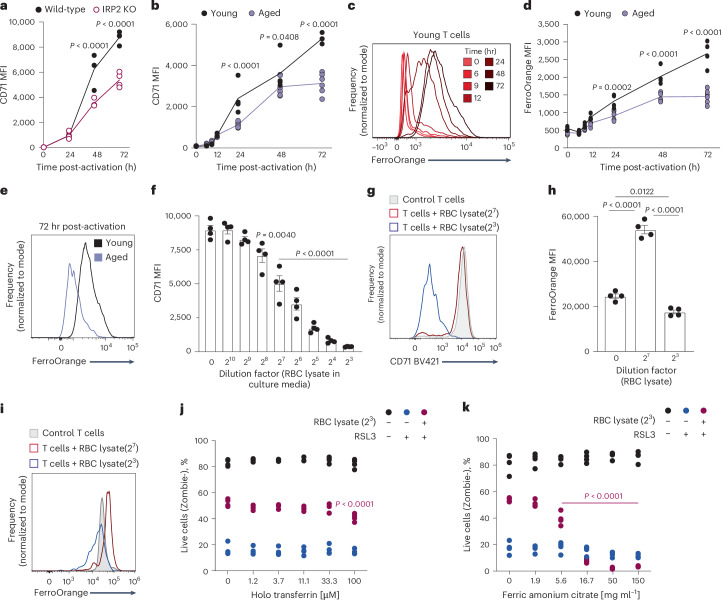
Exposure to hemolytic conditions suppresses CD71 and iron uptake during T cell activation to avoid ferroptosis. **a**,**b**, CD71 expression was analyzed at the indicated times during T cell activation, comparing (**a**) IRP2 wild-type (*n* = 4) versus KO T cells (*n* = 4) and (**b**) young (*n* = 4) versus aged (*n* = 6) T cells. **c**–**e**, Kinetic changes in labile iron (measured by FerroOrange) during activation in (**c**) young T cells and (**d,e**) young (*n* = 4) versus aged (*n* = 6) cells. Young T cells (*n* = 4) were activated ex vivo in media containing varying doses of RBC lysate. **f**–**i**, CD71 expression (**f,g**) and labile iron levels (**h,i**) were assessed by flow cytometry. Young T cells (*n* = 4) were activated in the presence of RSL3 ± RBC lysate (1:8 dilution), and cell viability was assessed following the addition of varying doses of holo-transferrin (**j**) or FAC (**k**; *P* values were calculated compared to 0 μM FAC/holo-transferrin within each condition). MFI was calculated by geometric mean. Bar graphs represent mean ± s.e.m. *P* values were calculated by one-way ANOVA (**f,h**) or two-way ANOVA (**a**,**b**,**d**,**j**,**k**) with Tukey’s or Sidak’s multiple comparisons tests. Each panel shows representative data of at least two independent experiments. Data points represent single mice. Source data

**Extended Data Fig. 6:**
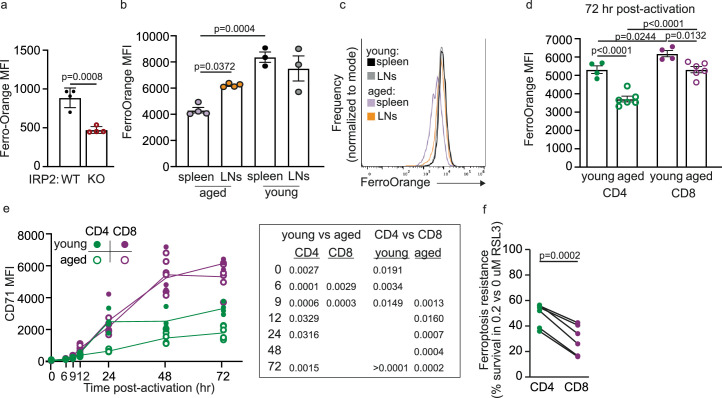
Enhanced ferroptosis resistance correlates with increased iron deficiency in aged CD4⁺ T cells. (**a**) Labile iron quantified using FerroOrange in IRP2 wild-type (n = 4) and KO (n = 4) T cells following activation. (**b**-**c**) T cells were purified from spleens and LNs derived from young (n = 3) and aged (n = 4) mice and activated for 72 hr followed by analysis of labile iron using FerroOrange. (**d**) Quantitation of labile iron using FerroOrange in young (n = 4) vs aged (n = 6) CD4^+^ and CD8^+^ T cells at 72 hr post-activation. (**e**) Activation-induced changes in CD71 expression in young (n = 4) and aged (n = 6) CD4^+^ and CD8^+^ T cells (same experiment shown in Fig. 7b now separately gating on CD4+ and CD8 + T cells). (**f**) Ferroptosis resistance calculated as the relative viability of T cells treated with 0.2μM vs 0μM RSL3, comparing aged (n = 6) CD4^+^ and CD8^+^ T cells. (Same experiment shown in Fig. 5b now separately gating on CD4^+^ and CD8^+^ T cells). Bar graphs represent mean ± SEM. Data points represent single mice. P values calculated by Two-way ANOVA with Sidak’s multiple comparison tests (**b**,**d**,**e**), or Two-tailed Student’s t-test (**a**, **f**). Source data

In young T cells, activation induced CD71, facilitating iron uptake^24^ (Fig. 7a). CD71 levels were reduced in IRP2-deficient T cells and aged T cells (Fig. 7a,b), suggesting that low IRP2 levels in aged T cells limits CD71 upregulation. To assess the impact on iron uptake, we used FerroOrange, a fluorescent probe that interacts with cellular ferrous ions^25^ (Fe^+2^). In young T cells, activation induced a robust increase in cellular iron (Fig. 7c). Iron levels were lower in aged T cells (Fig. 7d,e), and young IRP2-deficient T cells (Extended Data Fig. 6a). Notably, iron levels were higher in aged T cells from LNs compared to those from spleens, in agreement with their reduced exposure to the aged spleen milieu (Extended Data Fig. 6b,c).

To directly link exposure to the hemolytic microenvironment with CD71 downregulation, reduced iron uptake and ferroptosis resistance, young T cells were activated in the presence of RBC lysate. CD71 levels showed a dose-dependent suppression, with high concentrations (1:8) of RBC lysate in culture media almost completely eliminating CD71 expression (Fig. 7f,g). In line with these results, although supplementation with a low dose of RBC lysate (1:32) during T cell activation increased cellular iron compared to control T cells, consistent with RBCs serving as an iron source, a higher concentration of RBC lysate (1:8) diminished iron uptake (Fig. 7h,i). T cells treated with high concentrations of RBC lysate (1:8) were resistant to RSL3-induced ferroptosis and were not affected by addition of holo-transferrin (Fig. 7j), reflecting their low CD71 levels. Notably, the addition of FAC, which does not depend on CD71 for cell entry, abrogated ferroptosis resistance and reduced viability (Fig. 7k).

Our proposed association between cellular iron levels and CD71 expression during aging and ferroptosis resistance was further demonstrated when comparing CD4^+^ and CD8^+^ T cells. Age-related reduction in labile iron pool during activation was more pronounced in aged CD4^+^ T cells (Extended Data Fig. 6d). In agreement, only aged CD4^+^ T cells showed reduced induction of CD71 upon activation (Extended Data Fig. 6e). Consistent with these findings, aged CD4^+^ T cells were more resistant to RSL3-induced ferroptosis compared to CD8^+^ T cells (Extended Data Fig. 6f).

### Iron supplementation enhanced antigen-specific T cell responses in aged mice

**Fig. 8:**
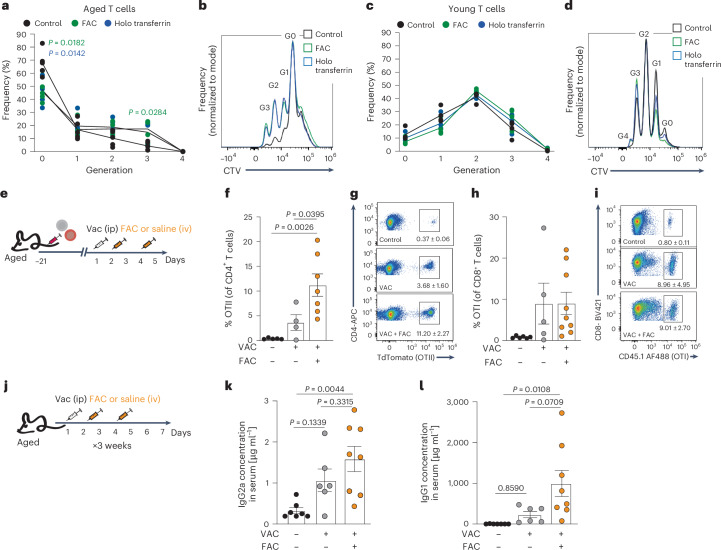
Iron supplementation enhanced antigen-specific T cell responses in aged mice. **a**–**d**, Proliferation analysis in aged (*n* = 5) and young (*n* = 4) T cells activated with and without supplementation of FAC or holo-transferrin. **e**, Experimental scheme. Aged C57Bl/6 wild-type mice were inoculated with transgenic T cells bearing known antigen specificity against ovalbumin (OVA). Each mouse was injected with a 1:1 mixture of OTII (TdTomato^+^CD4^+^) and OTI (CD45.1^+^CD8^+^) T cells. After 3 weeks, recipient mice were vaccinated i.p. with OVA/Alum adjuvant. Control mice received saline. On days 1 and 3 post-vaccination, vaccinated mice were infused with FAC or saline. The mice were sacrificed on day 5 post-vaccination and T cell content in the spleen was analyzed. **f**,**g**, Quantitation of OTII^+^ T cells (control: *n* = 5; vaccine: *n* = 4; vaccine+ FAC: *n* = 7). **h**,**i**, Quantitation of OTI^+^ T cells (control: *n* = 6; vaccine: *n* = 5; vaccine + FAC: *n* = 9). **j**, Scheme depicting experimental design: aged mice were vaccinated i.p. with OVA/Alum adjuvant. Control mice received saline. On days 1 and 3 post-vaccination, mice were infused with FAC or saline. This protocol was repeated three times before serum collection. **k**,**l**, OVA-specific IgG2a (**k**) and IgG1 (**l**) antibody titers in serum were quantified by ELISA (control: *n* = 7; vaccine: *n* = 6; vaccine + FAC: *n* = 8). Bar graphs represent mean ± s.e.m. Data points represent single mice. *P* values were calculated by one-way ANOVA with Sidak’s multiple comparisons test (**f**,**h**,**k**,**l**) or two-way repeated measure ANOVA with Tukey’s multiple comparison’s test (**a**,**c**). Source data

**Extended Data Fig. 7:**
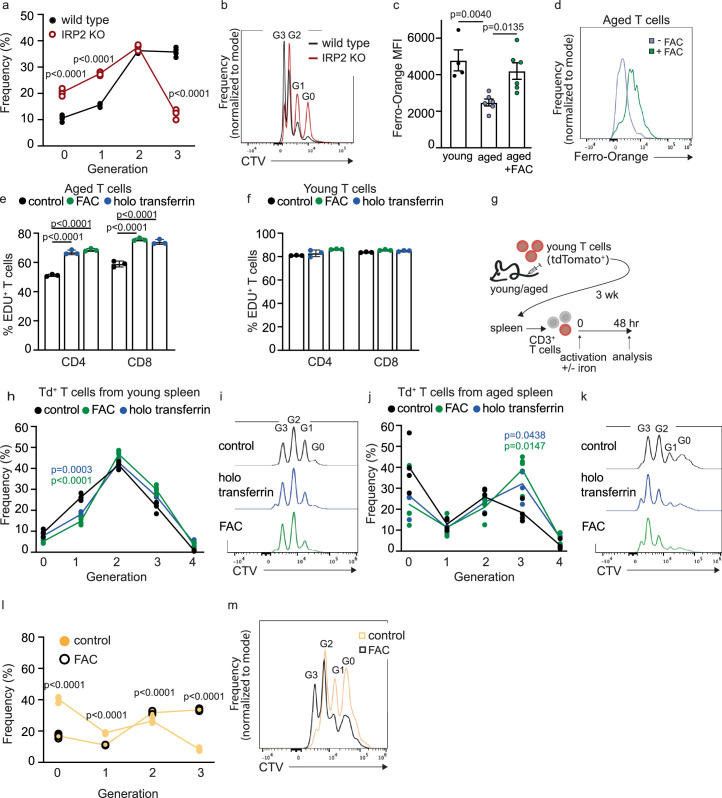
Iron supplementation restores activation-induced proliferation in aged T cells and young T Cells exposed to the aged spleen environment. (**a**,**b**) IRP2 wild-type (n = 4) and KO (n = 4) T cells were loaded with CellTrace violet, and activated to quantify proliferation. Gn indicates number of cell divisions. (**c**,**d**) FerroOrange fluorescence intensity in young (n = 4) and aged (n = 6) T cells, and aged T cells supplemented with ferric ammonium citrate (FAC). (**e**,**f**) Quantification of EDU incorporation in (**e**) aged (n = 3), and (**f**) young (n = 3) T cells during activation, with and without supplementation of FAC or holo-transferrin. (**g**) Scheme showing experimental design. Young T cells derived from TdTomato^+^ transgenic mice were transfused into young or aged C57Bl/6 wild-type recipients. After 3 weeks, recipient mice were sacrificed, and CD3^+^ T cells from spleens were activated ex vivo to analyze proliferation with and without iron supplementation. (**h**,**i**) Proliferation in TdTomato^+^ T cells derived from young spleens (n = 4). (**j**,**k**) Proliferation of TdTomato^+^ T cells derived from aged spleens (n = 4). (**l**,**m**) T cells were isolated from LNs of aged mice (n = a pool of 4 mice) and activated with and without iron supplementation (FAC) to assess proliferation using CTV. Gn represents the number of cell divisions. MFI: Mean Fluorescence Intensity, calculated by geometric mean. Bar graphs represent mean ± SEM. P values calculated by One-way ANOVA (**c**,**e**,**f**) or Two-way repeated measure ANOVA (**a**,**h**,**j**,**l**) with Sidak’s multiple comparisons test. Each panel shows representative data from at least 2 independent experiments. Data points represent single mice, except for (**l**). Source data

Iron is essential for DNA synthesis and cell proliferation. IRP2-deficient T cells showed reduced proliferation (Extended Data Fig. 7a,b), similar to aged T cells (Fig. 1). We hypothesized that functional iron deficiency contributes to reduced proliferation in aged T cells and tested whether iron supplementation could reverse this effect. Supplementation replenished cellular iron pool, as indicated by an enhanced FerroOrange signal (Extended Data Fig. 7c,d). To assess the impact of iron levels on proliferation, young and aged T cells were stimulated in the presence of FAC or holo-transferrin. Both treatments promoted DNA synthesis (Extended Data Fig. 7e) and proliferation in activated aged T cells, reducing the frequency of non-proliferating T cells (generation 0) and increasing the proportion of highly proliferating T cells (generation 3) (Fig. 8a,b). This effect was not observed in young T cells (Extended Data Fig. 7f and Fig. 8c,d). Similarly, iron supplementation enhanced proliferation in young T cells isolated from aged spleens following adoptive transfer (Extended Data Fig. 7g–k), consistent with their increased resistance to ferroptosis (Fig. 5l). Notably, iron supplementation further elevated proliferation also in aged T cells isolated from LNs (Extended Data Fig. 7l,m).

Vaccination responses are diminished in aged individuals^1^. The connection between iron availability and the T cell response to vaccination was confirmed in a recent study using hepcidin-induced iron deficiency showing that the T cell response to vaccination was diminished in mice treated with hepcidin and rescued by in vivo administration of FAC^26^. We took a similar approach to test whether iron supplementation could improve in vivo T cell responses in aged mice. T cells with a known antigen specificity against ovalbumin (OVA) were isolated from young transgenic mice and injected into aged recipients. Each mouse received equal amounts of OVA-specific OTI (CD8^+^CD45.1^+^) and OTII (CD4^+^TdTomato^+^) T cells, administered intravenously. Recipient mice were vaccinated intraperitoneally with OVA emulsified in alum adjuvant 3 weeks following cell transfer. Control mice were injected with saline. FAC was administered to vaccinated mice intravenously on days 1 and 3 following vaccination. Mice were euthanized on day 5, and T cell content in the spleen was analyzed by flow cytometry (Fig. 8e). Iron supplementation following vaccination increased the frequency of antigen-specific CD4^+^ T cells (OTII; Fig. 8f,g). The frequency of antigen-specific CD8^+^ T cells was not significantly affected by FAC administration (OTI; Fig. 8h,i), in agreement with our data showing that these cells were less prone to iron deficiency. These data encouraged us to investigate whether iron supplementation could affect the outcome of vaccination in aged mice, by quantifying antibody titers. Aged mice received three rounds of vaccination with OVA emulsified in Alum adjuvant, followed by transfusion with either FAC or saline at 24 and 72 h after vaccination. Serum was collected on day 21, and IgG1 and IgG2a titers were measured by ELISA (Fig. 8j). As expected from the use of Alum adjuvant, which promotes IgG1 class switching, Ova-specific IgG1 concentrations in vaccinated animals were approximately three orders of magnitude higher than OVA-specific IgG2a, which was barely detectable. For both antibody isotypes, only the combination of vaccination and iron supplementation significantly increased serum antibody levels compared to unvaccinated controls (Fig. 8k,l). Notably, for IgG1, iron supplementation improved antibody production relative to vaccination alone, although this did not reach statistical significance (*P* = 0.07; Fig. 8l). Thus, replenishing the labile iron pool in aged mice enhanced adaptive immune response to vaccination against a model antigen.

## Discussion

Aging manifests as a heterogeneous process among individuals and across distinct tissues within the same individual. Aging in different tissues could differ in pace and propensity, and includes alterations in tissue structure and cellularity, changes in transcription profile, and in the proteome^27–29^. Immune cells, including T lymphocytes, must adapt to changes in their immediate microenvironment as they populate different tissues. Such adaptation is manifested by changes in the cells’ metabolic phenotype, as T cells reshape their metabolism depending on the specific conditions and available resources in their host tissue. These include ad hoc adaptations at a site of inflammation or within a solid tumor, and homeostatic adaptations of tissue-resident lymphocytes to the specific conditions in their host tissue^30^. Our study shows how aging processes within the spleen shape the T cell aging trajectory. We demonstrate that exposure to signals originating from the aged spleen microenvironment, including the products of uncontrolled lysis of RBCs, induces an adaptive response, enabling T cells to survive this hostile milieu and resist ferroptosis by downregulating IRP2 and restricting labile iron pool. Iron supplementation abolishes this acquired ferroptosis resistance. However, this adaptation comes at a cost, impairing T cell functionality due to iron insufficiency. Iron administration enhances T cell responses in aged mice.

Acute ex vivo exposure to the aged spleen milieu or to RBC lysate kills a large portion of T cells. The higher the concentration, the smaller the portion of T cells surviving. Those that survive are resistant to ferroptosis, but are functionally impaired. Ferroptosis resistance in aged T cells is mediated, in part, through restriction of labile iron pool, by suppressing CD71 and upregulating ferritin- a mechanism consistent with findings in other cell types^31,32^. We demonstrate that ferroptosis resistance is mediated, in part, by downregulation of IRP2. IRP2 expression in aged T cells was reduced by 50%, yet aged T cells shared many functional similarities with IRP2 KO T cells. Consistently, partial suppression of IRP2 has been shown to reduce CD71 and increase ferritin levels^33^.

Other potential mechanisms contributing to ferroptosis resistance under hemolytic conditions could include the release from RBCs of redox-regulating enzymes (such as peroxiredoxin 2, glutathione peroxidase, catalase, and superoxide dismutase), and small molecule antioxidants (like α-tocopherol, glutathione, and ascorbic acid)^34^. RBCs also contain high concentrations of IL-33 (ref. ^35^). When released into the microenvironment, IL-33 could induce ferroptosis resistance through the ATF3/SLC7A11 axis^36^. Thus, hemolytic conditions originating in the aged spleen expose lymphocytes to toxic levels of heme and iron deposits while inducing protective mechanisms to promote survival, a form of adaptive homeostasis to a hostile microenvironment.

Hemolytic conditions in the aged spleen also release ATP into the microenvironment^37–39^. Extracellular ATP serves as a danger signal, indicative of tissue damage, and induces proinflammatory signals, such as the inflammasome^40^. CD39, together with CD73 and CD26, mediates the breakdown of ATP to adenosine, which inhibits T cell proliferation^41^. This pathway plays a central role in mediating immunosuppression within the tumor microenvironment^42^. CD39 is considered a marker of T cell dysfunction and senescence^9^. We identified exposure to the milieu of the aged spleen as the upstream signal inducing CD39 overexpression in aged T cells. Moreover, although previous studies suggested that elevation of CD39 was mediated by the byproducts of heme degradation, bilirubin^43^ and CO^44^, our use of an HO-1 inhibitor indicated that heme itself is a potent inducer of CD39 in aged T cells. Heme-mediated CD39 expression may represent another adaptive response of T cells to the hostile microenvironment, counteracting inflammatory signals in the aged spleen and, together with iron restriction, suppressing aged T cell proliferation.

Our proteomic analysis of pure naive T cells from young and aged mice revealed that cells from aged spleens are enriched with proteins associated with cellular stress, immune activation and inflammation. Many of these overexpressed proteins remained significantly elevated in aged T cells compared to young T cells, even after activation. These include proteins involved in protein maturation and folding, stress responses, inflammation and T cell effector functions. Our findings support a sustained loss of proteostasis in aged T cells, consistent with studies reporting the accumulation of misfolded proteins^45,46^, reduced activity of proteolytic systems such as the proteasome^47^ and autophagy^48^, and altered chaperone expression^49^. Further evidence for proteostasis dysregulation in aged T cells comes from their increased cell size^50^. One possible mechanism is oxidative stress, potentially induced by heme and iron within the aged spleen environment, as previous studies linked ROS to proteostasis-associated alterations in aged cells^51,52^.

Current interventions to rejuvenate T cell functions target various components of the proteostasis network, including inducers of autophagy and lysosomal activity such as mTOR inhibitors^53,54^, metformin^55,56^ and spermidine^48^. These interventions have been shown to enhance T cell responses and reduce inflammation. However, our findings suggest that some alterations in proteostasis may represent adaptative responses to the aged microenvironment. Consequently, restoring these pathways could expose T cells to harmful signals. For example, we found that developing resistance to ferroptosis is crucial for T cell survival in the aged spleen. Moreover, despite elevated intracellular ferritin levels, aged T cells remain iron deficient, suggesting an impaired ability to release iron via ferritinophagy, a selective form of autophagy. By avoiding ferritinophagy, aged T cells may protect themselves from ferroptosis, a mechanism previously described in senescent cells^57^. Therefore, combining proteostasis-enhancing therapies with interventions that mitigate iron overload and heme accumulation in the aged spleen could help prevent ferroptosis-driven lymphocyte loss. Alternatively, iron supplementation should be precisely timed to periods of physiological need (for example, vaccination) to avoid exacerbating toxicity.

Notably, our analysis of iron supplementation focused on its effects on T cell proliferation and antibody production following vaccination. To better inform vaccination strategies in older adults, further studies are needed to assess its impact on additional aspects of the immune response, including T cell differentiation, germinal center formation and B cell function. Moreover, given the proliferative benefit observed in LN T cells, the effects of systemic iron on LNs response to vaccination warrant further investigation.

Heme functions as the reactive center for various metal-based proteins but exhibits toxicity in its free form due to its hydrophobic nature and the presence of an iron atom, which catalyzes ROS production via the Fenton reaction. The byproducts of heme degradation—carbon monoxide (CO), biliverdin, and bilirubin—exert dual effects. Although they exhibit antioxidant properties, their accumulation can lead to cellular damage and apoptosis. Among these, bilirubin and CO have been specifically studied in T cells, where they were shown to inhibit T cell activation and enhance the suppressive capacity of regulatory T cells^17,18^. In this study, we focused on the direct effects of heme on T cells. Nonetheless, elevated levels of enzymes involved in heme catabolism in aged T cells, along with increased bilirubin and iron levels in aged spleens, suggest that the byproducts of heme degradation may also modulate T cell function within the aged spleen microenvironment.

Heme scavenging is mediated by various factors, including hemopexin, lipoproteins and serum albumin^16^. Among these, hemopexin has the highest affinity for heme and is currently being explored as a treatment for hemolytic diseases^58^. Our findings indicate that the microenvironment in aged spleens is inherently hemolytic, suggesting that therapy with heme scavengers might improve immune function in older individuals. Here, we used serum albumin to mitigate the toxic effects of heme. Although less efficient than hemopexin in binding heme, albumin could sequester many additional compounds harmful to T cells within the aged spleen milieu, including bilirubin^59^, ATP^60^ and iron. Furthermore, albumin could protect T cells from ROS-induced ferroptosis by increasing cellular cysteine levels^61^. Ex vivo, albumin efficiently protected young T cells exposed to heme or to interstitial fluids from aged spleens; however, it did not restore functionality in aged T cells. Rather, the only intervention that enhanced aged T cell functions was iron supplementation during activation, improving nucleotide biosynthesis and proliferation. Our findings reveal the hemolytic microenvironment that develops in spleens during aging as a systemic driver of multiple phenotypes characteristic of T cell aging.

## Methods

All experiments included in this study were pre-approved by the Technion’s Institutional Animal Care and Use Committee and comply with relevant ethical regulations.

### Mice

Young (7–10 weeks old) and aged (21-23 months old) C57BL/6JOlaHsd female mice were purchased from Envigo (Israel). For aging experiments, 8-month-old retired breeders were maintained for additional 13 to 15 months. Transgenic C57Bl/6 Rosa26^tdTomato/+^OTII mice were kindly provided by Z. Shulman (The Weizmann Institute of Science). C57Bl/6.SJLPtprcaPep3b;Ly5.1-Tg(TcraTcrb)1100Mjb/J (CD45.1 OTI) transgenic mice were kindly provided by M. Berger (The Hebrew University). C57Bl/6 Irp2^−/−^ (Ireb2^−/−^) mice were provided by T. Rouault (Molecular Medicine Program, National Institute of Child Health and Human Development, National Institutes of Health) to E. G. Meyron-Holtz. All mice were housed in specific pathogen-free conditions at the Technion Pre–Clinical Research Authority, an ALAC-accredited facility, and used in accordance with animal care guidelines from the Institutional Animal Care and Use Committee.

### T cell isolation and culture

Cells were obtained from spleens and LNs (inguinal, axillary, brachial, mandibular and jejunal) and either treated with RBC lysis buffer (Hybri-Max; Sigma-Aldrich, R7757) or directly purified by magnetic separation to obtain bulk CD3^+^ T cells or CD3^+^CD4^+^ T cells or naive (CD3^+^CD4^+^CD62L^+^CD44^lo^CD25^−^) T cells, using commercially available kits (STEMCELL Technologies: 19851, 19852, 19765). To purify naive T cells from aged mice, biotinylated anti-mouse/human CD44 (103004, BioLegend) and biotinylated anti-mouse CD25 (130-049-701, Miltenyi Biotech) were added to the commercial antibody cocktail, followed by a positive selection of CD62L^+^ naive T cells on a magnetic column. Primary T cells were cultured at 37 °C and 5% CO_2_ in RPMI supplemented with 10% FBS, 10 mM HEPES, penicillin/streptomycin and 0.004% beta-mercaptoethanol. For activation, T cells were cultured on plates precoated with anti-CD3 (2 μg ml^−1^; BioXcell, BE0001-1) and anti-CD28 (4 μg ml^−1^; BioXcell, BE0015-1). Resting cells were supplemented with 5 ng ml^−1^ recombinant murine IL-7 (Peprotech, 217-17-10). At indicated experiments, T cell cultures were supplemented with hemin chloride (Cayman, 16487), (1S,3 R)-RSL3 (Cayman, 19228), DFO (Cayman, 14595), Tin Mesoporphyrin IX (SnMP, Cayman, 19071), bovine serum albumin (BSA; Sigma-Aldrich, A9647), human holo-Transferrin (Sigma-Aldrich, T0665), human apo-Transferrin (Sigma-Aldrich, T2036), *N*-acetyl-L-cysteine (Sigma-Aldrich, A9165), Ferrostatin-1 (Sigma-Aldrich, SML05983), Liproxstatin-1 (Sigma-Aldrich, SML1414), Chlorazol Black/ Ferristatin II (Sigma-Aldrich, C1144), ammonium iron (III) citrate (Thermo-Scientific, A11199.30), interstitial fluid-enriched fraction from mouse spleen or RBC lysate.

### Adoptive T cell transfer

Young T cells were isolated from spleens of young C57Bl/6 Rosa26^tdTomato/+^ or C57Bl/6 Rosa26^tdTomato/+^OTII or CD45.1 OTI transgenic mice (8–12 weeks old). 5 M cells were inoculated by tail vein injection into wild-type C57Bl/6 young or aged recipients.

### Vaccination and in vivo iron supplementation

Aged mice were injected intraperitoneally with 100 µg OVA albumin (Vac-Stova, InvivoGen) or 0.9% saline, adsorbed in 40% Alum adjuvant (Alu-Gel-S, SERVA). On days 1 and 4 after vaccination, select mice received intravenous ferric ammonium citrate (900 µg per mouse; ThermoFisher). For antibody analysis, this protocol was repeated for three consecutive weeks before serum collection. For T cell proliferation analysis, young T cells were isolated from Rosa26^tdTomato/+^OTII or CD45.1 OTI transgenic mice, and mixed at a 1:1 ratio. A total of 5 M cells were injected via the tail vein into wild-type C57Bl/6 aged recipients. A single round of vaccination and ferric ammonium citrate administration was performed three weeks after adoptive transfer.

### FTY720 administration

FTY720 (Sigma, SML0700) was prepared and administered as previously described^62^at 1 mg kg^−1^ body weight i.p. for 2 weeks. In some experiments, adoptive transfer of transgenic TdTomato^+^ T cells was performed on day 1, together with the first FTY720 injection.

### Splenectomy

Splenectomy was performed under 2% isoflurane anesthesia, with preoperative administration of slow-release buprenorphine (0.05 ml, subcutaneously). The surgical site was shaved, disinfected with iodine and 70% ethanol, and ophthalmic ointment was applied. A vertical 6–8 mm incision was made in the left abdominal wall, and the spleen was carefully excised following vessel cauterization. The abdominal wall was closed with absorbable sutures, and the skin was closed with either sutures or wound clips. Mice were monitored postoperatively and allowed to recover for 2 weeks before adoptive cell transfer.

### Collection of spleen interstitial fluid-enriched fraction (SE)

Spleens from young or aged mice were gently dissociated on a 70μM cell strainer, in 5 ml PBS or RPMI, followed by a 5 min centrifugation at 385 *g* to separate cell pellet from SE fraction.

### RBC lysis

Whole blood was collected from mice via cardiac puncture into MiniCollect EDTA tubes (Greiner, 450531), diluted 1:1 in PBS, and subjected to leukoreduction using Histopaque (Sigma, 10771) at 400 *g*, without brake, for 30 min at room temperature. Obtained RBC pellets were lysed by 4 freeze/thaw cycles.

### Flow cytometry

For cell-surface staining, T cells were suspended in a separation buffer (PBS containing 2 % FBS and 2 mM EDTA) and incubated for 20 min, on ice. For intracellular staining, True-Nuclear Transcription Factor Buffer Set was used (BioLegend, 424401). For analysis of cytokine production, T cells underwent chemical stimulation (3 h; Cell Activation Cocktail 1:500; BioLegend, 423303). Cell viability was quantified by Zombie Violet or Zombie NIR Fixable Viability Kits (BioLegend, 423114 or 423106). Cell proliferation was assessed using CellTrace Violet Cell Proliferation Kit (Invitrogen, C34557). Staining for ferrous iron was done by incubation with 1 µM of FerroOrange (dojindo) in HBSS at 37 °C for 30 min in 5% CO_2_. Lipid peroxidation was assessed using BODIPY 581/591 C11 (Invitrogen), 5 µM in PBS at 37 °C 5% CO_2_ for 30 min, or Liperfluo (dojindo) following manufacturer’s protocol. Intracellular ROS was measured using by DCFDA/H2DCFDA Cellular ROS Assay Kit (Abcam) following manufacturer’s protocol. All data were collected on the Attune NxT Flow Cytometer (ThermoFisher) and analyzed using FlowJo (BD).

### EdU incorporation assay

Cells were incubated with 10 μM EdU (5-ethynyl-2′-deoxyuridine, Invitrogen, A10044) for 30 min at 37 °C in 5% CO_2_. Cells were then fixed with 4% paraformaldehyde for 10 minutes at room temperature, followed by permeabilization with 0.5% Triton X-100 in PBS for 10 min. EdU incorporation was detected using a click chemistry reaction, incubating cells with staining buffer containing 2 mM CuSO_4_, 100 mM freshly prepared sodium ascorbate, and 1 μM Alexa Fluor 647 azide (Invitrogen, A10277) in PBS for 30 min at room temperature. Cells were washed three times with PBS and either processed for blocking and antibody staining or directly acquired.

The following antibodies were used in this study: Alexa Fluor 647-AffiniPure Donkey Anti-Rabbit IgG (H + L) (711-605-152) and Alexa Fluor 488 AffiniPure Goat Anti-Rabbit IgG (H + L) (111-545-144) (both from Jackson); Alexa Fluor 488 anti-mouse CD45.1 (110717), Alexa Fluor 647 anti-mouse CD39 (143807), Alexa Fluor 647 anti-human/mouse Granzyme B (396421), Alexa Fluor 647 anti-mouse IL-10 (505014), Alexa Fluor 700 anti-mouse CD62L (104426), APC anti-mouse CD4 (100412), APC anti-mouse CD71 (113819), APC anti-mouse CD8a (100712), Brilliant Violet 421 anti-mouse CD3 (100227), Brilliant Violet 421 anti-mouse CD4 (100438), Brilliant Violet 421 anti-mouse CD71 (113813), Brilliant Violet 421™ anti-mouse CD8a (100737), Brilliant Violet 421 anti-mouse IFN-γ (505830), Brilliant Violet 510 anti-mouse CD4 (100449), Brilliant Violet 510 anti-mouse/human CD44 (103043), Brilliant Violet 510 anti-mouse CD69 (104531), FITC anti-mouse CD25 (102005), FITC anti-mouse CD28 (122007), FITC anti-mouse CD3 (100203), FITC anti-mouse CD4 (100406), FITC anti-mouse CD8a (100706), FITC anti-mouse CD95 (152605), FITC anti-mouse/human KLRG1 (138410), Pacific Blue anti-mouse/human CD45R/B220 (103230), PE anti-mouse CD19 (152407), PE anti-mouse CD25 (102007); PE anti-mouse CD3 (100206); PE anti-mouse CD39 (143804); PE anti-mouse CD4 (100407); PE anti-mouse CD45.1 (110707), PE anti-mouse CD69 (104508), PE anti-mouse IL-2 (503807), PerCP anti-mouse CD4 (100537), PerCP anti-mouse CD45 (103129) and purified anti-mouse CD16/32 (101302) (all from BioLegend); recombinant rabbit anti-ferritin (FTH1) (ab75973) and recombinant rabbit anti-heme oxygenase 1 (HO-1) (ab52947) (both from Abcam).

### Untargeted, whole-cell proteomics

For sample preparation, naive T cells (CD4^+^CD62L^hi^CD44^lo^CD25^−^) were purified from spleens of young and aged mice and were either frozen or activated for 24 h.

For proteolysis, proteins were extracted in 9 M urea, 400 mM ammonium bicarbonate and 10 mM DTT following two cycles of sonication, reduced with 3 mM DTT (60 °C for 30 min), modified with 9 mM iodoacetamide in 400 mM ammonium bicarbonate (in the dark, room temperature for 30 min) and digested in 1 M urea, 50 mM ammonium bicarbonate with modified trypsin (Promega) at a 1:50 enzyme-to-substrate ratio, overnight at 37 °C. An additional second trypsinization was done for 4 h.

Mass spectrometry analysis. Tryptic peptides were desalted using C18 stagetips, dried and resuspended in 0.1% Formic acid. Peptides were resolved by reverse-phase chromatography on 0.075 ×180-mm fused silica capillaries (J&W) packed with Reprosil reversed phase material (Dr Maisch GmbH, Germany). The peptides were eluted with different concentration of Acetonitrile with 0.1% of formic acid: a linear 180 min gradient of 5 to 28% acetonitrile followed by a 15 min gradient of 28% to 95% and 25 min at 95% acetonitrile with 0.1% formic acid in water at flow rates of 0.15 μl min^−1^. Mass spectrometry was performed by Q Executive HFX mass spectrometer (ThermoFisher) in a positive mode (m/z 350–1,200, resolution 120,000 for MS1 and 15,000 for MS2) using repetitively full MS scan followed by collision induced dissociation (HCD, at 27 normalized collision energy) of the 30 most dominant ions (>1 charge) selected from the first MS scan. The AGC settings were 3 × 10^6^ for the full MS and 1 × 10^5^ for the MS/MS scans. A dynamic exclusion list was enabled with exclusion duration of 20 s. The mass spectrometry data were analyzed using the MaxQuant 1.5.2.8 software^63^ for peak picking and identification using the Andromeda search engine, searching against the mouse proteome from the Uniprot database with mass tolerance of 6 ppm for the precursor masses and the fragment ions. Oxidation on methionine and protein N-terminus acetylation were accepted as variable modifications and carbamidomethyl on cysteine was accepted as static modifications. Minimal peptide length was set to seven amino acids and a maximum of two miscleavages was allowed. The data was quantified by label-free analysis using the same software. Peptide- and protein-level false discovery rates were filtered to 1% using the target-decoy strategy. Protein tables were filtered to eliminate the identifications from the reverse database, and common contaminants and single peptide identifications. The data was quantified by label-free analysis using the same software. Statistical analysis of the identification and quantization results was done using Perseus 1.6.2.2 software^64^. Gene set enrichment analysis was performed using the GSEA software (https://www.gsea-msigdb.org/gsea/index.jsp)^65,66^.

### Histology

Spleens were fixed with 4% PFA, processed (Leica TP1020), paraffin embedded and sectioned (4 mm; Leica RM2265 Rotary Microtome). Sections were stretched on a warm 37 °C water bath, collected into slides, and dried at 37 °C overnight. Sections were processed for H&E and Prussian blue staining. Slides were scanned by 3DHistech Panoramic 250 Flash III and visualized using the CaseViewer software (3DHISTECH).

### Immunohistochemistry

Tissue dissection, fixation, embedding and sectioning was performed as previously described^67^, followed by staining with AF647 anti-CD169 (BioLegend) and DAPI. Imaging was performed using an LSM710 AxioObserver microscope and ZEN software (Zeiss).

### OVA-specific IgG1 and IgG2a ELISA

96-well plates were coated overnight at 4 °C with 50 μg ml^−1^ OVA (diluted in 50 mM sodium carbonate/bicarbonate buffer, pH 9.6). Plates were washed with PBST (PBS containing 0.05% Tween 20) and blocked with 3% BSA in PBS for 1 h at room temperature. Serum samples (diluted in PBST), controls and standards were added and incubated for 2 h. Following washes, plates were incubated with either anti-mouse IgG1 (rat anti-mouse IgG1-HRP;1:5,000) or anti-mouse IgG2a (rat anti-mouse IgG2a-HRP; 1:2,500) antibodies in PBST for 30 min. TMB substrate was added, and absorbance at 650 nm was measured using CLARIO Star Plus using Smart Control MARS Version 4.10 (BMG Labtech).

### Immunoblot

T cells were lysed with RIPA buffer (sigma R0278) containing protease inhibitor cocktail) on ice for 15 min and centrifuged at 14,000 *g* for 15 min, 4 °C. Equal amounts of protein samples were separated on 12% SDS–PAGE and transferred to a 0.45 μm PVDF membrane. Membranes were blocked with 5% BSA in TBST buffer and probed with the indicated primary antibody overnight at 4 °C. Membranes were then washed and incubated with HRP-goat anti-rabbit IgG (H + L; Jackson ImmunoResearch; 111-035-144) for 1 h at room temperature, followed by incubation with ECL substrate (Cyanagen). Images were obtained using Fusion Pulse and EvolutionCapt pulse 6 software (Vilber Lourmat). Primary antibodies included homemade rabbit anti mouse IRP2 (provided by T. Rouault, Molecular Medicine Program, National Institute of Child Health and Human Development, National Institutes of Health to E.Meyron-Holtz) and rabbit anti-ACTA1 (Sigma-Aldrich, A2066).

### Heme and bilirubin quantitation

Spleen extract (50 µl) was mixed with 200 µl heme reagent (Heme Assay Kit, Sigma-Aldrich, MAK316), or with 200 µl total/direct bilirubin reagent (Bilirubin Assay Kit, Sigma-Aldrich, MAK126) and incubated for 5 to 10 min. Absorption was measured at 400 nm (heme) or 530 nm (bilirubin) on a plate reader (Biotek). Reads were normalized to total cell numbers. For intracellular heme content, 5 M cells were lysed in 60 µl RIPA buffer (Sigma-Aldrich, R0278), followed by three freeze/thaw cycles. Lysates were centrifuged for 15 min at 11,000 *g* at 4 °C. Then, 50 µl cell lysates were mixed with 200 µl heme reagent, and absorption was measured.

### Quantitative, real-time PCR

Total RNA was extracted from CD3^+^ T cells using QuickRNA Micro prep Kit (ZYMO RESEARCH, R1050). cDNA was synthesized using the High-Capacity cDNA RT kit (Applied Biosystems, 4374966). Quantitative PCR was run on QuantStudio 3 Real-Time PCR System, 96-well (Applied Biosystems) using Fast SYBR Green Master Mix (Applied Biosystems). The following primers were used: *Blvra*: forward (F): 5’-AAGATCCCGAACCTCTCTCT-3’, reverse (R): 5’-TTATCAAGGCTCCCAAGTTCTC-3’; *Blvrb*: F: 5’-AAGCTGTCATCGTGCTACTG-3’, R: 5’-CAGTTAGTGGTTGGTCTCCTATG-3’; *Fth1*: F: 5’-TCAACCGCCAGATCAACC-3’, R: 5’-TCAGTTTCTCGGCATGCTC-3’; *Ftl*: 5’-CGTGGATCTGTGTCTTGCTTCA-3’, R: 5’-GCGAAGAGACGGTGCAGACT-3’; *Ho-1*: F: 5’-GTTCAAACAGCTCTATCGTGC-3’, R: 5’-TCTTTGTGTTCCTCTGTCAGC-3’; *Rps18*: F: 5’-CCGCCATGTCTCTAGTGATCC-3’, R: 5’-GGTGAGGTCGATGTCTGCTT-3’.

### Statistics and reproducibility

Sample sizes of three to nine animals were determined based on prior experience to ensure adequate statistical power. Larger sample sizes were consistently used for aged mice compared to young mice to account for greater interindividual variability and higher mortality rates in older animals. No statistical methods were used to predetermine sample sizes, but our sample sizes are similar to those reported in previous publications^11^. Exclusion criteria were defined in advance, and aged mice exhibiting splenomegaly (suggestive of underlying clinical conditions) were excluded from analysis. All reported findings were successfully replicated. Mice were randomly assigned to in vivo experimental groups. Data collection and analysis were not blinded to experimental conditions, except for immunohistochemistry data collection and the analysis of vaccination responses, which involved only aged mice. Statistical analyses were performed using Prism (GraphPad Software). Specific statistical tests are detailed in the figure legends. All tests were two sided. Data distribution was assumed to be normal, but this was not formally tested. For analyses comparing T cells derived from spleens and LNs of young and aged mice, only biologically meaningful comparisons were included in the multiple comparisons test, namely comparisons between spleens and LNs within the same age group, and comparisons of each organ across age groups.

### Reporting summary

Further information on research design is available in the Nature Portfolio Reporting Summary linked to this article.

## Supplementary information

Reporting Summary

Supplementary Data 1 Proteomics raw data.

## Source data

Source Data Fig. 1 Statistical source data Fig. 1.

Source Data Fig. 2 Statistical source data Fig. 2.

Source Data Fig. 3 Statistical source data Fig. 3.

Source Data Fig. 3 Uncropped images – for Fig. 3.

Source Data Fig. 4 Statistical source data Fig. 4.

Source Data Fig. 5 Statistical source data Fig. 5.

Source Data Fig. 6 Statistical source data Fig. 6.

Source Data Fig. 6 Uncropped blots- Fig. 6.

Source Data Fig. 7 Statistical source data Fig. 7.

Source Data Fig. 8 Statistical source data Fig. 8.

Source Data Extended Data Fig. 1 Statistical source data Extended Data Fig. 1.

Source Data Extended Data Fig. 2 Statistical source data Extended Data Fig. 2.

Source Data Extended Data Fig. 3 Statistical source data Extended Data Fig. 3.

Source Data Extended Data Fig. 4 Statistical source data Extended Data Fig. 4.

Source Data Extended Data Fig. 5 Statistical source data Extended Data Fig. 5.

Source Data Extended Data Fig. 6 Statistical source data Extended Data Fig. 6.

Source Data Extended Data Fig. 7 Statistical source data Extended Data Fig. 7.

## Data Availability

All data supporting the study’s findings are provided in the Source Data and Supplementary Information, including statistical source data for main figures and extended data figures, uncropped images (related to Fig. 3) and uncropped western blots (related to Fig.6). The mass spectrometry proteomics data have been deposited to the ProteomeXchange Consortium via the PRIDE^68^ partner repository with the dataset identifier PXD067102. Source data are provided with this paper.

## References

[1] Mittelbrunn, M. & Kroemer, G. Hallmarks of T cell aging. *Nat. Immunol.* **22**, 687–698 (2021). 33986548 10.1038/s41590-021-00927-z

[2] Lopez-Otin, C., Blasco, M. A., Partridge, L., Serrano, M. & Kroemer, G. Hallmarks of aging: an expanding universe. *Cell* **186**, 243–278 (2023). 36599349 10.1016/j.cell.2022.11.001

[3] Yousefzadeh, M. J. et al. An aged immune system drives senescence and ageing of solid organs. *Nature* **594**, 100–105 (2021). 33981041 10.1038/s41586-021-03547-7 PMC8684299

[4] Desdin-Mico, G. et al. T cells with dysfunctional mitochondria induce multimorbidity and premature senescence. *Science* **368**, 1371–1376 (2020). 32439659 10.1126/science.aax0860 PMC7616968

[5] Bronte, V. & Pittet, M. J. The spleen in local and systemic regulation of immunity. *Immunity* **39**, 806–818 (2013). 24238338 10.1016/j.immuni.2013.10.010 PMC3912742

[6] Slusarczyk, P., et al. Impaired iron recycling from erythrocytes is an early hallmark of aging. *Elife* **12**, e79196 (2023). 36719185 10.7554/eLife.79196 PMC9931393

[7] Yang, W. S. & Stockwell, B. R. Ferroptosis: death by lipid peroxidation. *Trends Cell Biol.* **26**, 165–176 (2016). 26653790 10.1016/j.tcb.2015.10.014 PMC4764384

[8] Rodriguez, I. J. et al. Immunosenescence study of T cells: a systematic review. *Front. Immunol.* **11**, 604591 (2020). 33519813 10.3389/fimmu.2020.604591 PMC7843425

[9] Fang, F. et al. Expression of CD39 on activated T cells impairs their survival in older individuals. *Cell Rep.* **14**, 1218–1231 (2016). 26832412 10.1016/j.celrep.2016.01.002 PMC4851554

[10] Chiba, K. et al. FTY720, a novel immunosuppressant, induces sequestration of circulating mature lymphocytes by acceleration of lymphocyte homing in rats. I. FTY720 selectively decreases the number of circulating mature lymphocytes by acceleration of lymphocyte homing. *J. Immunol.* **160**, 5037–5044 (1998). 9590253

[11] Ron-Harel, N. et al. Defective respiration and one-carbon metabolism contribute to impaired naive T cell activation in aged mice. *Proc. Natl Acad. Sci. USA.* **115**, 13347–13352 (2018). 30530686 10.1073/pnas.1804149115 PMC6310842

[12] Han, S., Georgiev, P., Ringel, A. E., Sharpe, A. H. & Haigis, M. C. Age-associated remodeling of T cell immunity and metabolism. *Cell Metab.* **35**, 36–55 (2023). 36473467 10.1016/j.cmet.2022.11.005 PMC10799654

[13] Turner, V. M. & Mabbott, N. A. Influence of ageing on the microarchitecture of the spleen and lymph nodes. *Biogerontology* **18**, 723–738 (2017). 28501894 10.1007/s10522-017-9707-7 PMC5597693

[14] Sullivan, S. G., Baysal, E. & Stern, A. Inhibition of hemin-induced hemolysis by desferrioxamine: binding of hemin to red cell membranes and the effects of alteration of membrane sulfhydryl groups. *Biochim. Biophys. Acta* **1104**, 38–44 (1992). 1550852 10.1016/0005-2736(92)90129-a

[15] Stout, D. L. The role of transferrin in heme transport. *Biochem. Biophys. Res. Commun.* **189**, 765–770 (1992). 1472048 10.1016/0006-291x(92)92267-2

[16] De Simone, S., et al. *Biomolecules* **13**, 575 (2023). 36979511 10.3390/biom13030575 PMC10046553

[17] Song, R. et al. Carbon monoxide inhibits T lymphocyte proliferation via caspase-dependent pathway. *J. Immunol.* **172**, 1220–1226 (2004). 14707100 10.4049/jimmunol.172.2.1220

[18] Yamashita, K. et al. Biliverdin, a natural product of heme catabolism, induces tolerance to cardiac allografts. *FASEB J.* **18**, 765–767 (2004). 14977878 10.1096/fj.03-0839fje

[19] Voltarelli, V. A., et al. Heme: the Lord of the Iron Ring. *Antioxidants (Basel)* **12**, 1074 (2023). 37237940 10.3390/antiox12051074 PMC10215292

[20] Yang, W. S. et al. Regulation of ferroptotic cancer cell death by GPX4. *Cell* **156**, 317–331 (2014). 24439385 10.1016/j.cell.2013.12.010 PMC4076414

[21] Yan, H. F. et al. Ferroptosis: mechanisms and links with diseases. *Signal Transduct. Target Ther.* **6**, 49 (2021). 33536413 10.1038/s41392-020-00428-9 PMC7858612

[22] Zhang, D. L., Ghosh, M. C. & Rouault, T. A. The physiological functions of iron regulatory proteins in iron homeostasis - an update. *Front Pharm.* **5**, 124 (2014). 10.3389/fphar.2014.00124 PMC4056636 24982634

[23] Moroishi, T., Nishiyama, M., Takeda, Y., Iwai, K. & Nakayama, K. I. The FBXL5-IRP2 axis is integral to control of iron metabolism in vivo. *Cell Metab.* **14**, 339–351 (2011). 21907140 10.1016/j.cmet.2011.07.011

[24] Jabara, H. H. et al. A missense mutation in TFRC, encoding transferrin receptor 1, causes combined immunodeficiency. *Nat. Genet.* **48**, 74–78 (2016). 26642240 10.1038/ng.3465 PMC4696875

[25] Yu, F. et al. Dynamic O-GlcNAcylation coordinates ferritinophagy and mitophagy to activate ferroptosis. *Cell Discov.* **8**, 40 (2022). 35504898 10.1038/s41421-022-00390-6 PMC9065108

[26] Frost, J. N. et al. Hepcidin-mediated hypoferremia disrupts immune responses to vaccination and infection. *Med (N. Y)* **2**, 164–179 e112 (2021). 10.1016/j.medj.2020.10.004 PMC7895906 33665641

[27] Oh, H. S. et al. Organ aging signatures in the plasma proteome track health and disease. *Nature* **624**, 164–172 (2023). 38057571 10.1038/s41586-023-06802-1 PMC10700136

[28] Nie, C. et al. Distinct biological ages of organs and systems identified from a multi-omics study. *Cell Rep.* **38**, 110459 (2022). 35263580 10.1016/j.celrep.2022.110459

[29] Kimmel, J. C. et al. Murine single-cell RNA-seq reveals cell-identity- and tissue-specific trajectories of aging. *Genome Res* **29**, 2088–2103 (2019). 31754020 10.1101/gr.253880.119 PMC6886498

[30] Varanasi, S. K., Kumar, S. V. & Rouse, B. T. Determinants of tissue-specific metabolic adaptation of T cells. *Cell Metab.* **32**, 908–919 (2020). 33181092 10.1016/j.cmet.2020.10.013 PMC7710599

[31] He, J. et al. Reprogramming of iron metabolism confers ferroptosis resistance in ECM-detached cells. *iScience* **26**, 106827 (2023). 37250802 10.1016/j.isci.2023.106827 PMC10209538

[32] Gao, M., Monian, P., Quadri, N., Ramasamy, R. & Jiang, X. Glutaminolysis and transferrin regulate ferroptosis. *Mol. Cell* **59**, 298–308 (2015). 26166707 10.1016/j.molcel.2015.06.011 PMC4506736

[33] Hwang, J. et al. Inhibition of IRP2-dependent reprogramming of iron metabolism suppresses tumor growth in colorectal cancer. *Cell Commun. Signal* **22**, 412 (2024). 39180081 10.1186/s12964-024-01769-6 PMC11342626

[34] Yildiz, D., Uslu, C., Cakir, Y. & Oztas, H. L. -Cysteine influx and efflux: a possible role for red blood cells in regulation of redox status of the plasma. *Free Radic. Res* **40**, 507–512 (2006). 16551577 10.1080/10715760600602902

[35] Wei, J. et al. Red blood cells store and release interleukin-33. *J. Investig. Med* **63**, 806–810 (2015). 26107423 10.1097/JIM.0000000000000213 PMC4767276

[36] Wu, Q. et al. Macrophages originated IL-33/ST2 inhibits ferroptosis in endometriosis via the ATF3/SLC7A11 axis. *Cell Death Dis.* **14**, 668 (2023). 37816731 10.1038/s41419-023-06182-4 PMC10564909

[37] Sikora, J., Orlov, S. N., Furuya, K. & Grygorczyk, R. Hemolysis is a primary ATP-release mechanism in human erythrocytes. *Blood* **124**, 2150–2157 (2014). 25097178 10.1182/blood-2014-05-572024 PMC4186543

[38] McMahon, T. J., Darrow, C. C., Hoehn, B. A. & Zhu, H. Generation and export of red blood cell ATP in health and disease. *Front. Physiol.* **12**, 754638 (2021). 34803737 10.3389/fphys.2021.754638 PMC8602689

[39] Ferguson, B. S. et al. Red blood cell ATP release correlates with red blood cell hemolysis. *Am. J. Physiol. Cell Physiol.* **321**, C761–C769 (2021). 34495762 10.1152/ajpcell.00510.2020

[40] Di Virgilio, F., Sarti, A. C. & Coutinho-Silva, R. Purinergic signaling, DAMPs, and inflammation. *Am. J. Physiol. Cell Physiol.* **318**, C832–C835 (2020). 32159362 10.1152/ajpcell.00053.2020

[41] Antonioli, L., Pacher, P., Vizi, E. S. & Hasko, G. CD39 and CD73 in immunity and inflammation. *Trends Mol. Med.* **19**, 355–367 (2013). 23601906 10.1016/j.molmed.2013.03.005 PMC3674206

[42] Xia, C., Yin, S., To, K. K. W. & Fu, L. CD39/CD73/A2AR pathway and cancer immunotherapy. *Mol. Cancer* **22**, 44 (2023). 36859386 10.1186/s12943-023-01733-x PMC9979453

[43] Longhi, M. S. et al. Bilirubin suppresses Th17 immunity in colitis by upregulating CD39. *JCI Insight*10.1172/jci.insight.92791 (2017). 10.1172/jci.insight.92791 PMC5414551 28469075

[44] Lee, G. R., Shaefi, S. & Otterbein, L. E. HO-1 and CD39: it takes two to protect the realm. *Front. Immunol.* **10**, 1765 (2019). 31402920 10.3389/fimmu.2019.01765 PMC6676250

[45] Cuanalo-Contreras, K. et al. Extensive accumulation of misfolded protein aggregates during natural aging and senescence. *Front. Aging Neurosci.* **14**, 1090109 (2022). 36778589 10.3389/fnagi.2022.1090109 PMC9909609

[46] Vilchez, D., Saez, I. & Dillin, A. The role of protein clearance mechanisms in organismal ageing and age-related diseases. *Nat. Commun.* **5**, 5659 (2014). 25482515 10.1038/ncomms6659

[47] Ponnappan, U., Zhong, M. & Trebilcock, G. U. Decreased proteasome-mediated degradation in T cells from the elderly: a role in immune senescence. *Cell Immunol.* **192**, 167–174 (1999). 10087185 10.1006/cimm.1998.1418

[48] Alsaleh, G. et al. Autophagy in T cells from aged donors is maintained by spermidine and correlates with function and vaccine responses. *Elife* **9**, e57950 (2020). 33317695 10.7554/eLife.57950 PMC7744099

[49] Valdor, R. et al. Chaperone-mediated autophagy regulates T cell responses through targeted degradation of negative regulators of T cell activation. *Nat. Immunol.* **15**, 1046–1054 (2014). 25263126 10.1038/ni.3003 PMC4208273

[50] Jin, J. et al. FOXO1 deficiency impairs proteostasis in aged T cells. *Sci. Adv.* **6**, eaba1808 (2020). 32494657 10.1126/sciadv.aba1808 PMC7176426

[51] Gressler, A. E., Leng, H., Zinecker, H. & Simon, A. K. Proteostasis in T cell aging. *Semin. Immunol.* **70**, 101838 (2023). 37708826 10.1016/j.smim.2023.101838 PMC10804938

[52] Korovila, I. et al. Proteostasis, oxidative stress and aging. *Redox Biol.* **13**, 550–567 (2017). 28763764 10.1016/j.redox.2017.07.008 PMC5536880

[53] Mannick, J. B. et al. mTOR inhibition improves immune function in the elderly. *Sci. Transl. Med.* **6**, 268ra179 (2014). 25540326 10.1126/scitranslmed.3009892

[54] Mannick, J. B. et al. TORC1 inhibition enhances immune function and reduces infections in the elderly. *Sci. Transl. Med.* **10**, eaaq1564 (2018). 29997249 10.1126/scitranslmed.aaq1564

[55] Bharath, L. P. et al. Metformin enhances autophagy and normalizes mitochondrial function to alleviate aging-associated inflammation. *Cell Metab.* **32**, 44–55 e46 (2020). 32402267 10.1016/j.cmet.2020.04.015 PMC7217133

[56] Yang, J. et al. The effect of metformin on senescence of T lymphocytes. *Immun. Ageing* **20**, 73 (2023). 38087369 10.1186/s12979-023-00394-0 PMC10714529

[57] Masaldan, S. et al. Iron accumulation in senescent cells is coupled with impaired ferritinophagy and inhibition of ferroptosis. *Redox Biol.* **14**, 100–115 (2018). 28888202 10.1016/j.redox.2017.08.015 PMC5596264

[58] Gentinetta, T. et al. Plasma-derived hemopexin as a candidate therapeutic agent for acute vaso-occlusion in sickle cell disease: preclinical evidence. *J. Clin. Med.* **11**, 630 (2022). 35160081 10.3390/jcm11030630 PMC8836474

[59] Zorzi, A., Linciano, S. & Angelini, A. Non-covalent albumin-binding ligands for extending the circulating half-life of small biotherapeutics. *Medchemcomm* **10**, 1068–1081 (2019). 31391879 10.1039/c9md00018f PMC6644573

[60] Bauer, M., Baumann, J. & Trommer, W. E. ATP binding to bovine serum albumin. *FEBS Lett.* **313**, 288–290 (1992). 1332884 10.1016/0014-5793(92)81211-4

[61] Armenta, D. A. et al. Ferroptosis inhibition by lysosome-dependent catabolism of extracellular protein. *Cell Chem. Biol.* **29**, 1588–1600 e1587 (2022). 36306785 10.1016/j.chembiol.2022.10.006 PMC9762237

[62] Hu, W. et al. Regulatory T cells function in established systemic inflammation and reverse fatal autoimmunity. *Nat. Immunol.* **22**, 1163–1174 (2021). 34426690 10.1038/s41590-021-01001-4 PMC9341271

[63] Cox, J. et al. Accurate proteome-wide label-free quantification by delayed normalization and maximal peptide ratio extraction, termed MaxLFQ. *Mol. Cell Proteom.* **13**, 2513–2526 (2014). 10.1074/mcp.M113.031591 PMC4159666 24942700

[64] Tyanova, S. et al. The Perseus computational platform for comprehensive analysis of (prote)omics data. *Nat. Methods* **13**, 731–740 (2016). 27348712 10.1038/nmeth.3901

[65] Subramanian, A. et al. Gene set enrichment analysis: a knowledge-based approach for interpreting genome-wide expression profiles. *Proc. Natl Acad. Sci. USA.* **102**, 15545–15550 (2005). 16199517 10.1073/pnas.0506580102 PMC1239896

[66] Mootha, V. K. et al. PGC-1alpha-responsive genes involved in oxidative phosphorylation are coordinately downregulated in human diabetes. *Nat. Genet.* **34**, 267–273 (2003). 12808457 10.1038/ng1180

[67] Fra-Bido, S., Walker, S. A., Innocentin, S. & Linterman, M. A. Optimized immunofluorescence staining protocol for imaging germinal centers in secondary lymphoid tissues of vaccinated mice. *STAR Protoc.* **2**, 100499 (2021). 34195671 10.1016/j.xpro.2021.100499 PMC8233161

[68] Perez-Riverol, Y. et al. The PRIDE database at 20 years: 2025 update. *Nucleic Acids Res.* **53**, D543–D553 (2025). 39494541 10.1093/nar/gkae1011 PMC11701690

