# Supplementary material for: Heme and iron toxicity in the aged spleen impairs T cell immunity through iron deprivation
Source: Nat Aging. 2025 Oct 17;5(11):2247–62. doi: 10.1038/s43587-025-00981-4 (PMC12618244; doi:10.1038/s43587-025-00981-4)
Supplement: Supplementary file 1 — Reporting Summary [file 43587_2025_981_MOESM1_ESM.pdf]

nature portfolio

Corresponding author(s): Noga Ron-Harel
Last updated by author(s): Aug 6, 2025

Reporting Summary

Nature Portfolio wishes to improve the reproducibility of the work that we publish. This form provides structure for consistency and transparency in reporting. For further information on Nature Portfolio policies, see our Editorial Policies and the Editorial Policy Checklist.

Statistics

For all statistical analyses, confirm that the following items are present in the figure legend, table legend, main text, or Methods section.

n/a	Confirmed	
☐	☒	The exact sample size (*n*) for each experimental group/condition, given as a discrete number and unit of measurement
☐	☒	A statement on whether measurements were taken from distinct samples or whether the same sample was measured repeatedly
☐	☒	The statistical test(s) used AND whether they are one- or two-sided<br>*Only common tests should be described solely by name; describe more complex techniques in the Methods section.*
☐	☒	A description of all covariates tested
☐	☒	A description of any assumptions or corrections, such as tests of normality and adjustment for multiple comparisons
☐	☒	A full description of the statistical parameters including central tendency (e.g. means) or other basic estimates (e.g. regression coefficient) AND variation (e.g. standard deviation) or associated estimates of uncertainty (e.g. confidence intervals)
☐	☒	For null hypothesis testing, the test statistic (e.g. *F*, *t*, *r*) with confidence intervals, effect sizes, degrees of freedom and *P* value noted<br>*Give P values as exact values whenever suitable.*
☒	☐	For Bayesian analysis, information on the choice of priors and Markov chain Monte Carlo settings
☒	☐	For hierarchical and complex designs, identification of the appropriate level for tests and full reporting of outcomes
☒	☐	Estimates of effect sizes (e.g. Cohen's *d*, Pearson's *r*), indicating how they were calculated

Our web collection on statistics for biologists contains articles on many of the points above.

Software and code

Policy information about availability of computer code

Data collection	QuantStudio design and analysis software (V1.5.2.8), Attune NxT software (V3.2.1), Xcalibur (ThermoFisher Scientific), Zen Black (Zeiss), EvolutionCapt pulse 6 software (Vilber Lourmat), Smart Control MARS Version 4.10 (BMG Labtech)
Data analysis	Maxquant (1.6.17.0), Perseus (1.6.2.2), FlowJo (10.10), SlideViewer (2.7), GraphPad Prism (10), SkylineTM Daily

For manuscripts utilizing custom algorithms or software that are central to the research but not yet described in published literature, software must be made available to editors and reviewers. We strongly encourage code deposition in a community repository (e.g. GitHub). See the Nature Portfolio guidelines for submitting code & software for further information.

Data

Policy information about availability of data

All manuscripts must include a data availability statement. This statement should provide the following information, where applicable:

- Accession codes, unique identifiers, or web links for publicly available datasets
- A description of any restrictions on data availability
- For clinical datasets or third party data, please ensure that the statement adheres to our policy

All relevant data was submitted with the manuscript. The mass spectrometry proteomics data have been deposited to the ProteomeXchange Consortium via the PRIDE partner repository with the dataset identifier PXD067102.

Research involving human participants, their data, or biological material

Policy information about studies with human participants or human data. See also policy information about sex, gender (identity/presentation), and sexual orientation and race, ethnicity and racism.

Reporting on sex and gender	*Use the terms sex (biological attribute) and gender (shaped by social and cultural circumstances) carefully in order to avoid confusing both terms. Indicate if findings apply to only one sex or gender; describe whether sex and gender were considered in study design; whether sex and/or gender was determined based on self-reporting or assigned and methods used. Provide in the source data disaggregated sex and gender data, where this information has been collected, and if consent has been obtained for sharing of individual-level data; provide overall numbers in this Reporting Summary. Please state if this information has not been collected. Report sex- and gender-based analyses where performed, justify reasons for lack of sex- and gender-based analysis.*
Reporting on race, ethnicity, or other socially relevant groupings	*Please specify the socially constructed or socially relevant categorization variable(s) used in your manuscript and explain why they were used. Please note that such variables should not be used as proxies for other socially constructed/relevant variables (for example, race or ethnicity should not be used as a proxy for socioeconomic status). Provide clear definitions of the relevant terms used, how they were provided (by the participants/respondents, the researchers, or third parties), and the method(s) used to classify people into the different categories (e.g. self-report, census or administrative data, social media data, etc.) Please provide details about how you controlled for confounding variables in your analyses.*
Population characteristics	*Describe the covariate-relevant population characteristics of the human research participants (e.g. age, genotypic information, past and current diagnosis and treatment categories). If you filled out the behavioural & social sciences study design questions and have nothing to add here, write "See above."*
Recruitment	*Describe how participants were recruited. Outline any potential self-selection bias or other biases that may be present and how these are likely to impact results.*
Ethics oversight	*Identify the organization(s) that approved the study protocol.*

Note that full information on the approval of the study protocol must also be provided in the manuscript.

Field-specific reporting

Please select the one below that is the best fit for your research. If you are not sure, read the appropriate sections before making your selection.

☒ Life sciences ☐ Behavioural & social sciences ☐ Ecological, evolutionary & environmental sciences

For a reference copy of the document with all sections, see nature.com/documents/nr-reporting-summary-flat.pdf

Life sciences study design

All studies must disclose on these points even when the disclosure is negative.

Sample size	Sample sizes were determined based on previous experience. Samples size for aged mice was always larger compared to young, to account for the larger variability across individuals. For in vivo studies, the sample size for aged mice was further increased to account for increased death rates at old age.
Data exclusions	Exclusion was done based on predetermined decisions, excluding aged mice that suffered spleenomegaly and showed a skewed T cell population, indicative of an underlying clinical condition.
Replication	All reported findings were successfully replicated, at least twice. The only experiment performed only once was LC/MS proteomics, whose findings were validated by other experimental methods, including qPCR and flow cytometry.
Randomization	Most of our experiments compared untreated young and aged mice. Randomization was irrelevant. For in vivo experiments (vaccination, FTY and splenectomy) , mice were randomly allocated into treatment groups.
Blinding	Most data in this manuscript were automatically recorded by flow cytometry and was therefore not subjected to subjective interpretation of the experimentalist. Blinding during sample collection and preparation of young and aged mice is largely impossible since the differences in spleen morphology and cellularity are very pronounced. Investigators were however blinded during data collection and analysis of immunohistochemistry, and during analysis of vaccination responses that included only aged mice.

Reporting for specific materials, systems and methods

We require information from authors about some types of materials, experimental systems and methods used in many studies. Here, indicate whether each material, system or method listed is relevant to your study. If you are not sure if a list item applies to your research, read the appropriate section before selecting a response.

Materials & experimental systems

n/a	Involved in the study
☐	☒ Antibodies
☒	☐ Eukaryotic cell lines
☒	☐ Palaeontology and archaeology
☐	☒ Animals and other organisms
☒	☐ Clinical data
☒	☐ Dual use research of concern
☒	☐ Plants

Methods

n/a	Involved in the study
☒	☐ ChIP-seq
☐	☒ Flow cytometry
☒	☐ MRI-based neuroimaging

Antibodies

Antibodies used

Alexa Fluor 647-AffiniPure Donkey Anti-Rabbit IgG (H+L) Jackson 711-605-152, Clone: polyclonal, 1:2000.

Alexa Fluor® 488 AffiniPure™ Goat Anti-Rabbit IgG (H+L) Jackson 111-545-144, Clone: polyclonal, 1:2000.

Alexa Fluor® 488 anti-mouse CD45.1 Biolegend 110717, Clone: A20, 1:200.

Alexa Fluor® 647 anti-mouse CD39 Biolegend 143807, Clone: Duha59, 1:200.

Alexa Fluor® 647 anti-human/mouse Granzyme B Biolegend 396421, Clone: QA18A28, 1:100.

Alexa Fluor® 647 anti-mouse IL-10 Biolegend 505014, Clone: JES5-16E3, 1:100.

Alexa Fluor® 700 anti-mouse CD62L Biolegend 104426, Clone: MEL-14, 1:200.

APC anti-mouse CD4 Biolegend 100412, Clone: GK1.5, 1:200. APC anti-mouse CD71 Biolegend 113819, Clone: RI7217, 1:200. APC anti-mouse CD8a Biolegend 100712, Clone: 53-6.7, 1:200. Brilliant Violet 421™ anti-mouse CD3 Biolegend 100227, Clone: 17A2, 1:200.

Brilliant Violet 421™ anti-mouse CD4 BioLegend 100438, Clone: GK1.5, 1:200.

Brilliant Violet 421™ anti-mouse CD71 Biolegend 113813, Clone: RI7217, 1:200. Brilliant Violet 421™ anti-mouse CD8a Biolegend 100737, Clone: 53-6.7, 1:200.

Brilliant Violet 421™ anti-mouse IFN-γ Biolegend 505830, Clone: XMG1.2, 1:100.

Brilliant Violet 510™ anti-mouse CD4 Biolegend 100449, Clone: GK1.5, 1:200.

Brilliant Violet 510™ anti-mouse/human CD44 Biolegend 103043, Clone: IM7, 1:200.

Brilliant Violet 510™ anti-mouse CD69 Biolegend 104531, Clone: H1.2F3, 1:200.

FITC anti-mouse CD25 Biolegend 102005, Clone: PC61, 1:200.

FITC anti-mouse CD28 Biolegend 122007, Clone: E18, 1:100. FITC anti-mouse CD3 Biolegend 100203, Clone: 17A2, 1:200.

FITC anti-mouse CD4 Biolegend 100406, Clone: GK1.5, 1:200. FITC anti-mouse CD8a Biolegend 100706, Clone: 53-6.7, 1:200.

FITC anti-mouse CD95 Biolegend 152605, Clone: SA367H8, 1:100.

FITC anti-mouse/human KLRG1 Biolegend 138410, Clone: 2F1/KLRG1, 1:100.

Pacific Blue™ anti-mouse/human CD45R/B220 Biolegend 103230, Clone RA3-6B2, 1:100.

PE anti-mouse CD19 Biolegend 152407, Clone: 1D3/CD19, 1:100.

PE anti-mouse CD25 Biolegend 102007, Clone PC61, 1:200. PE anti-mouse CD3 Biolegend 100206, Clone: 17A2, 1:200.

PE anti-mouse CD39 Biolegend 143804, Clone: Duha59, 1:200.

PE anti-mouse CD4 Biolegend 100407, Clone: GK1.5, 1:200.

PE anti-mouse CD45.1 Biolegend 110707, Clone: A20, 1:200.

PE anti-mouse CD69 Biolegend 104508, Clone: H1.2F3, 1:200.

PE anti-mouse IL-2 Biolegend 503807, Clone JES6-5H4, 1:100.

PerCP anti-mouse CD4 Biolegend 100537, Clone: RM4-5, 1:200.

PerCP anti-mouse CD45 Biolegend 103129, Clone: 30-F11, 1:200.

Purified anti-mouse CD16/32 Biolegend 101302, Clone 93, 1:100.

Recombinant Rabbit Anti-Ferritin (FTH1) Abcam ab75973, Clone: EPR3004Y, 1:100.

Recombinant Rabbit Anti-Heme Oxygenase 1(HO-1) Abcam ab52947, Clone: EP1391Y, 1:100.

Validation

All antibodies used in this study were pre-validated by the manufacturers for the applications used in this study.

Animals and other research organisms

Policy information about studies involving animals; ARRIVE guidelines recommended for reporting animal research, and Sex and Gender in Research

Laboratory animals

C57BL/6JOlaHsd Females. 7-10 weeks old, and 20-23 months old.
C57Bl/6 Rosa26tdTomato/+OTII 7-10 weeks old
C57Bl/6.SJLPtprcaPep3b;Ly5.1-Tg(TcraTcrb)1100Mjb/J (CD45.1 OTI) 7-10 weeks old
C57Bl/6 Irp2–/– (Ireb2tm1Roua) mice
Mice were house under a 12hr light/dark cycle, with ambient temp. kept on 20-23 deg, and 30-70% humidity.
Mice were fed Ad-lib, with Altromin 1324 pellets.

Wild animals

The study did not involve wild animals

Reporting on sex

This study was performed on young and aged F mice.

Field-collected samples

The study did not involve samples collected from the field

Ethics oversight

Technion Institutional Animal Care and Use Committee.

Note that full information on the approval of the study protocol must also be provided in the manuscript.

Plants

Seed stocks

Report on the source of all seed stocks or other plant material used. If applicable, state the seed stock centre and catalogue number. If plant specimens were collected from the field, describe the collection location, date and sampling procedures.

Novel plant genotypes

Describe the methods by which all novel plant genotypes were produced. This includes those generated by transgenic approaches, gene editing, chemical/radiation-based mutagenesis and hybridization. For transgenic lines, describe the transformation method, the number of independent lines analyzed and the generation upon which experiments were performed. For gene-edited lines, describe the editor used, the endogenous sequence targeted for editing, the targeting guide RNA sequence (if applicable) and how the editor was applied.

Authentication

Describe any authentication procedures for each seed stock used or novel genotype generated. Describe any experiments used to assess the effect of a mutation and, where applicable, how potential secondary effects (e.g. second site T-DNA insertions, mosiacism, off-target gene editing) were examined.

Flow Cytometry

Plots

Confirm that:

☒ The axis labels state the marker and fluorochrome used (e.g. CD4-FITC).

☒ The axis scales are clearly visible. Include numbers along axes only for bottom left plot of group (a 'group' is an analysis of identical markers).

☒ All plots are contour plots with outliers or pseudocolor plots.

☒ A numerical value for number of cells or percentage (with statistics) is provided.

Methodology

Sample preparation

For cell-surface staining, T cells were suspended in a separation buffer (PBS containing 2 % FBS and 2 mM EDTA) and incubated for 20 min, on ice. For intracellular staining, True-Nuclear™ Transcription Factor Buffer Set was used (Biolegend, 424401). For analysis of cytokine production, T cells underwent chemical stimulation (3 hrs; Cell Activation Cocktail 1:500; Biolegend, 423303). Cell viability was quantified by Zombie Violet™ or Zombie NIR™ Fixable Viability Kits (Biolegend, 423114 or 423106). Cell proliferation was assessed using CellTrace™ Violet Cell Proliferation Kit (Invitrogen, C34557). Staining for ferrous iron was done by incubation with 1µM of FerroOrange (dojindo) in HBSS at 37°C for 30 minutes in 5% CO_2. Lipid peroxidation was assessed using BODIPY™ 581/591 C11 (Invitrogen), 5 µM in PBS at 37°C 5% CO_2 for 30 minutes, or Liperfluo (dojindo) following manufacturer's protocol. Intracellular ROS was measured using by DCFDA / H2DCFDA - Cellular ROS Assay

	Kit (Abcam) following manufacturer's protocol. All data were collected on the Attune NxT Flow Cytometer (Thermo Fisher) and analyzed using FlowJo (BD).
Instrument	AttuneNxT 4 lasers
Software	Collection: Attune NxT software (V3.2.1)<br>Analysis: FlowJo (BD; Version 10.10)
Cell population abundance	Sorting was used for isolating naive T cells. Population purity was >90%
Gating strategy	Our gating strategy starts with a FSC/SSC plot to gate on the lymphocytes and exclude cell debri, followed by exclusion of doublets (FSCA/FSCW plot), and live/dead discrimination (using ZombiNIR or Zombie Violet). positive gates were experimentally determined based on unstained controls, FMO and biological controls when necessary.

☒ Tick this box to confirm that a figure exemplifying the gating strategy is provided in the Supplementary Information.
