# Supplementary figures and images for: Heme and iron toxicity in the aged spleen impairs T cell immunity through iron deprivation
Source: Nat Aging. 2025 Oct 17;5(11):2247–62. doi: 10.1038/s43587-025-00981-4 (PMC12618244; doi:10.1038/s43587-025-00981-4)

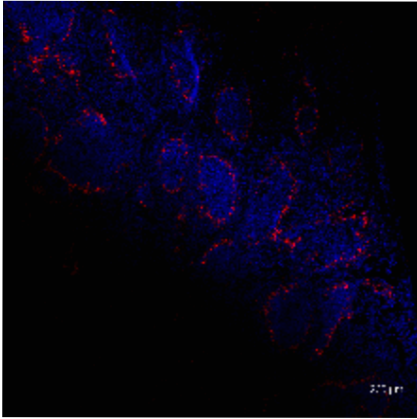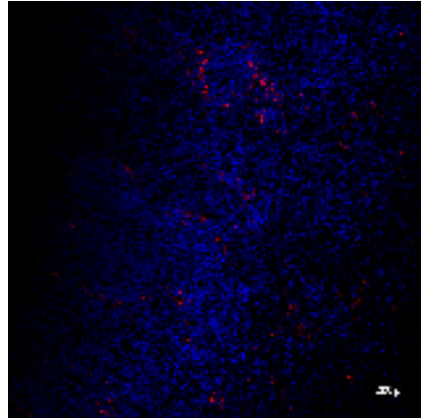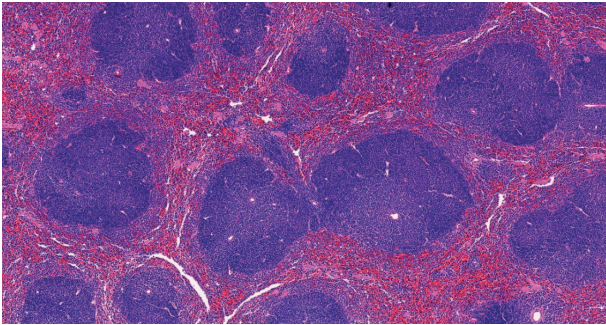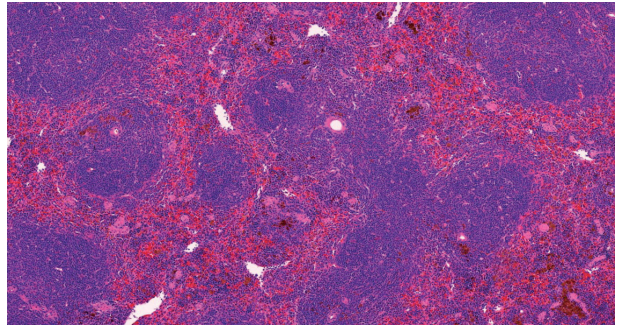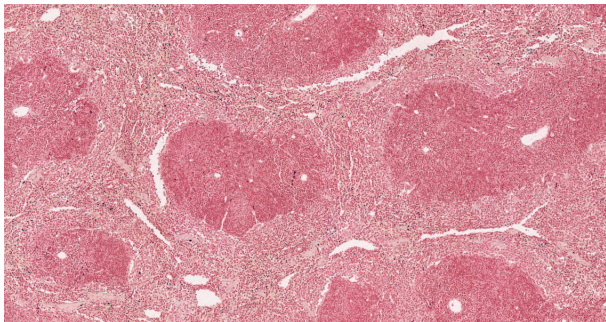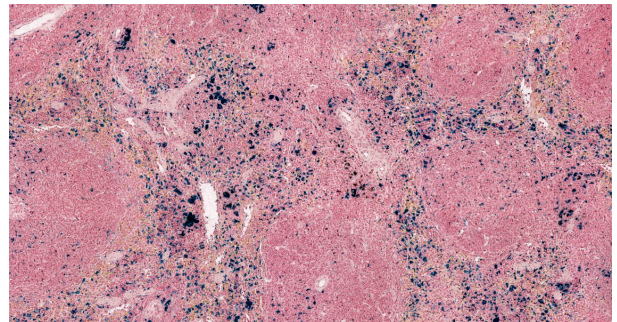

Supplement: Supplementary file 6 — Uncropped images – for Fig. 3. [file 43587_2025_981_MOESM6_ESM.pdf]

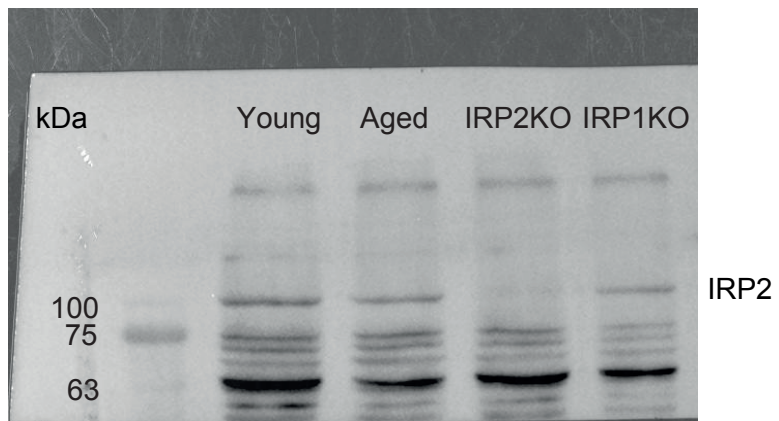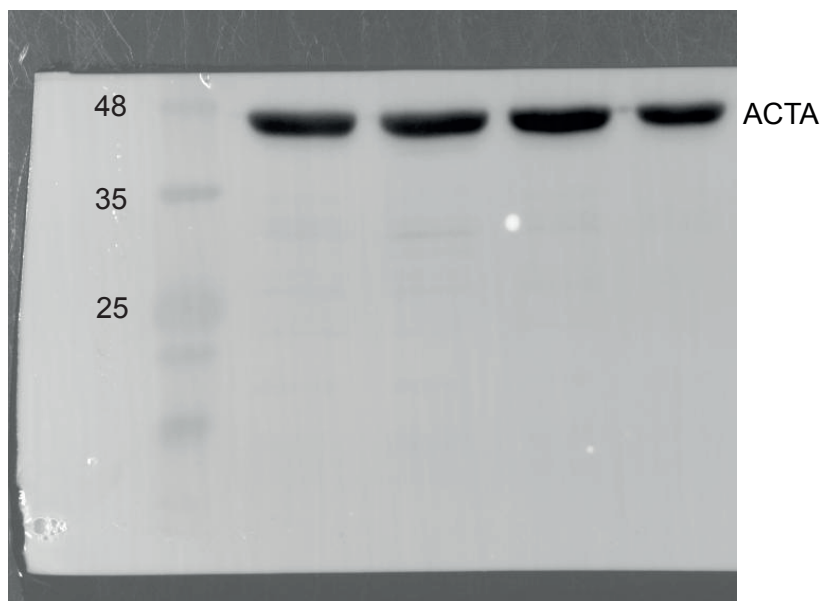

Supplement: Supplementary file 10 — Uncropped blots- Fig. 6. [file 43587_2025_981_MOESM10_ESM.pdf]
